# Cystathionine-β-synthase: Molecular Regulation and Pharmacological Inhibition

**DOI:** 10.3390/biom10050697

**Published:** 2020-04-30

**Authors:** Karim Zuhra, Fiona Augsburger, Tomas Majtan, Csaba Szabo

**Affiliations:** 1 Chair of Pharmacology, Section of Medicine, University of Fribourg, 1702 Fribourg, Switzerland; karim.zuhra@unifr.ch (K.Z.); fiona.augsburger@unifr.ch (F.A.); 2 Department of Pediatrics, University of Colorado Anschutz Medical Campus, Aurora, CO 80045, USA; tomas.majtan@cuanschutz.edu

**Keywords:** hydrogen sulfide, cancer, Down syndrome, pharmacology, homocysteine, cystathionine

## Abstract

Cystathionine-β-synthase (CBS), the first (and rate-limiting) enzyme in the transsulfuration pathway, is an important mammalian enzyme in health and disease. Its biochemical functions under physiological conditions include the metabolism of homocysteine (a cytotoxic molecule and cardiovascular risk factor) and the generation of hydrogen sulfide (H_2_S), a gaseous biological mediator with multiple regulatory roles in the vascular, nervous, and immune system. CBS is up-regulated in several diseases, including Down syndrome and many forms of cancer; in these conditions, the preclinical data indicate that inhibition or inactivation of CBS exerts beneficial effects. This article overviews the current information on the expression, tissue distribution, physiological roles, and biochemistry of CBS, followed by a comprehensive overview of direct and indirect approaches to inhibit the enzyme. Among the small-molecule CBS inhibitors, the review highlights the specificity and selectivity problems related to many of the commonly used “CBS inhibitors” (e.g., aminooxyacetic acid) and provides a comprehensive review of their pharmacological actions under physiological conditions and in various disease models.

## 1. CBS: Discovery, Regulation, and Physiological Roles

### 1.1. CBS: Discovery and Early Studies 

The transsulfuration pathway is an important metabolic pathway in which the interconversion of cysteine and homocysteine occurs through the intermediate cystathionine. We distinguish two transsulfuration pathways: the “forward transsulfuration pathway” (the bacterial pathway, which involves the transfer of the thiol group from cysteine to homocysteine) and the “reverse transsulfuration pathway” (the mammalian pathway, which involves the transfer of the thiol group from homocysteine to cysteine). Transsulfuration was originally discovered by Vincent du Vigneaud in the 1930s and 1940s. His work (which started at Washington University, and continued at Cornell University) focused on the oxidation of sulfur-containing amino acids in various mammalian tissues (and subsequently in live animals as well). It was du Vigneaud who realized that a mammalian metabolic pathway involving the interconversion of cysteine and homocysteine exists; he was also the scientist who discovered and named the intermediate of the reaction: cystathionine. Du Vigneaud initially simply termed the process as “transsulfuration” [1]; subsequently the terminology has been revised such that “transsulfuration” is now used to describe the bacterial system and “reverse transsulfuration” is the official term for the mammalian process. However, for simplicity, in the current article we will use term “transsulfuration” to designate the mammalian system of homocysteine to cysteine conversion. 

Importantly, during his studies focusing on the interconversion of sulfur-containing amino acids, du Vigneaud (together with Francis Birkley) published a paper in 1942, which also noted the formation of the gas hydrogen sulfide (H_2_S) from some of these reactions [2]. In this experiment, liver homogenates were used to measure the formation of cysteine from homocysteine and serine, and H_2_S formation was noted as a side reaction. However, in du Vigneaud’s time, the field of biochemistry was not advanced enough to identify specific enzymes responsible for these reactions. Moreover, the discovery that transsulfuration is associated with the biogenesis of H_2_S was not followed up further (neither by the du Vigneaud group, nor by others); the field had to wait many decades for the appreciation of the biological importance of this reaction.

**Figure 1:**
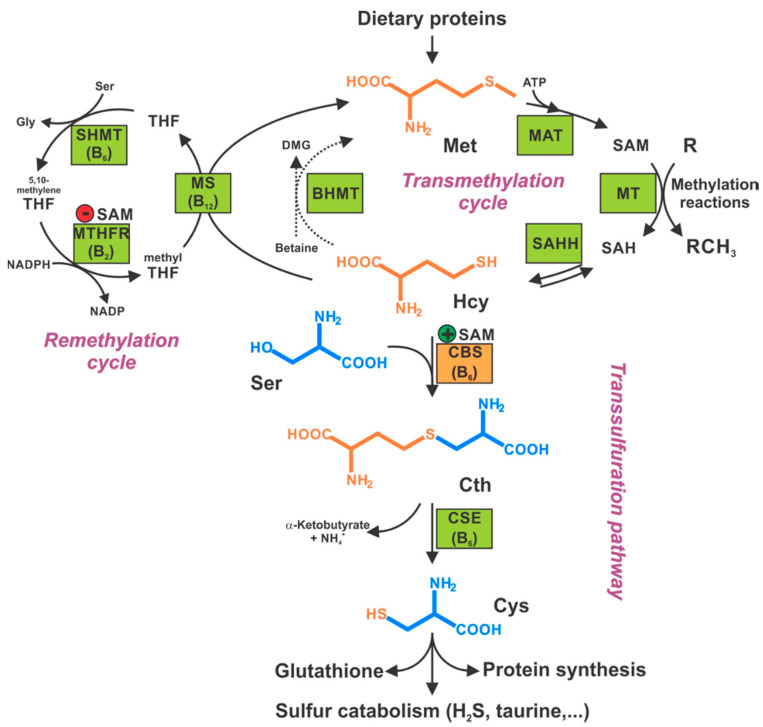
Role of CBS and other enzymes in the regulation of mammalian sulfur amino acid metabolism. Methionine (Met), an essential amino acid taken from dietary proteins, is condensed with ATP by methionine adenosyltransferase (MAT) to form S-adenosylmethionine (SAM). SAM serves as a universal methyl donor for multiple methylation reactions catalyzed by various methyltransferases (MT) yielding methylated product and S-adenosylhomocysteine (SAH). SAH is subsequently hydrolyzed by SAH hydrolase (SAHH) into adenosine and homocysteine (Hcy). Hcy is then distributed between two competing pathways. To conserve Met, Hcy is remethylated back to Met by the action of either liver-dependent betaine homocysteine methyltransferase (BHMT) or ubiquitous methionine synthase (MS) using betaine and methyl tetrahydrofolate (methyl-THF), respectively, as the methyl donor. To generate Cys, Hcy is irreversible diverted from the methionine cycle to the transsulfuration pathway by cystathionine beta-synthase (CBS)-catalyzed condensation with serine (Ser) forming cystathionine (Cth), which is subsequently hydrolyzed by cystathionine gamma-lyase (CSE, an enzyme also referred to as “CGL” in the literature) into cysteine (Cys). Importantly, SAM regulates the flux of Hcy through the competing pathways by allosteric activation of CBS and inhibition of methylenetetrahydrofolate reductase (MTHFR). Interestingly, all enzymes of the transsulfuration pathway and the remethylation (folate cycle) enzymes require assistance of a member of vitamin B family: B2 (riboflavin) in MTHFR, B6 (pyridoxine) in serine hydroxymethyltransferase (SHMT), CBS and CSE, B9 (folic acid) as a one-carbon carrier of the remethylation cycle, or B12 (cobalamin) in MS.

The various enzyme(s) involved in transsulfuration reactions have been identified in the late 1960s through the discovery of several different reactions these enzymes catalyze (see below). Cystathionine-**β**-synthase (CBS) is the first (and rate-limiting) enzyme in the transsulfuration pathway. The multiple enzymatic processes CBS catalyzes were gradually discovered by multiple investigators [3,4,5,6,7,8,9,10]; these reactions (see also below) are also illustrated by the multiple names the enzyme had in the early years—such as **β**-thionase, cysteine synthase, L-serine hydro-lyase (adding homocysteine), methylcysteine synthase, and serine sulfhydrase. In fact, one of the current official names of CBS is “L-serine hydro-lyase (adding homocysteine; L-cystathionine-forming)”. The current understanding of the role of CBS in mammalian sulfur amino acid metabolism (as well as the cooperative role of the other H_2_S producing enzymes) [11] is depicted in Figure 1.

In 1988, Kraus and colleagues mapped the human *CBS* gene to chromosome 21q22.3 [12]. Subsequently, the same group has cloned and sequenced the entire human *CBS* gene [13]. Starting in the same time period, and continuing to the present day, the fine details of CBS biochemistry and molecular biology have been identified, and the physiological and pathophysiological roles of this enzyme have been characterized (see below). 

### 1.2. The Molecular Organization of Human CBS

**Figure 2:**
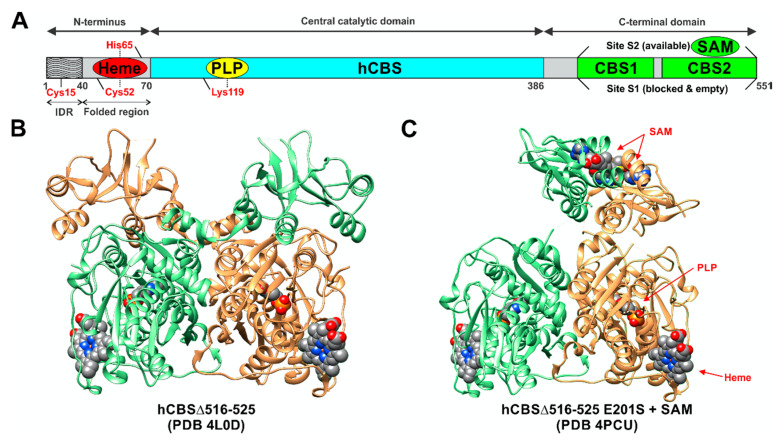
Domain organization and structure of hCBS. (**A**) Human CBS consists of three architectural regions. The N-terminal domain spanning residues 1–70 contains two distinct regions. The first 40 residues constitute intrinsically disordered region (IDR) with residue Cys15 playing role in transient heme-binding and protein aggregation. Residues 40–70 form a folded region, which binds heme cofactor, axially ligated by residues Cys52 and His65. A conserved catalytic core, covering residues 70–386, contains the PLP cofactor, where the catalysis occurs. In the resting state, the PLP forms an internal aldimine intermediate via the Schiff base bond with the ε-amino group of Lys119. The C-terminal regulatory domain spanning residues 386–551 contains a flexible linker followed by a tandem of CBS domains (CBS1 and CBS2), which form binding clefts for SAM housing. However, the site S1 is blocked by bulky hydrophobic residues, while the site S2 is available and can bind SAM, which activates the enzyme. B, C: Crystal structures of engineered human CBS in SAM-free basal (**B**) and SAM-bound activated (**C**) conformations. Note that crystal structures of human CBS are only available for its engineered hCBSΔ516–525 construct lacking a loop consisting of 10 amino acid residues from the C-terminal regulatory domain. Catalytically, the construct is identical to a full-length native enzyme; however, it forms dimers rather than tetramers or higher order oligomers typical for the full-length CBS. Two subunits in each dimer are depicted in light green and orange. Cofactors (heme, PLP, SAM) are shown in spheres.

**Figure 3:**
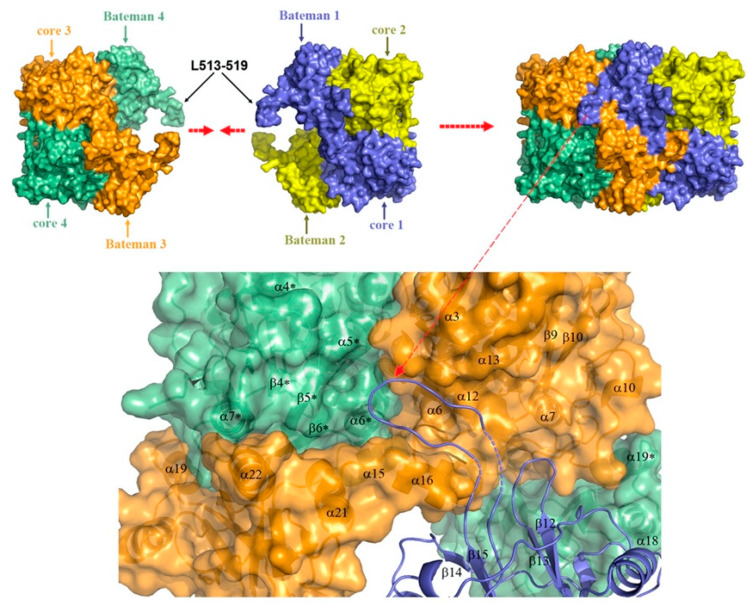
A proposed model of hCBS tetramerization. The tetramerization of hCBS is sustained by the interactions of each Bateman module (the C-terminal regulatory domain) with the Bateman modules and the catalytic cores of the complementary dimer. The tetramer is stabilized by interactions between loop 513–529, which serves as a “hook” locking the two dimers together, and the residues located at the cavity formed by the helices α6, α12, α15, and α16. Asterisks designate secondary structure elements to one of the two subunits in the dimer (orange—no symbol; green—*). Reproduced by permission [16].

Human CBS is a tetramer of 63-kDa subunits (Figure 2 and Figure 3). Each subunit binds, in addition to its two substrates (homocysteine and serine) three additional ligands: pyridoxal-5′-phosphate (PLP, the active form of vitamin B6), forming a Schiff base with Lys119, S-adenosylmethionine (SAM; also known as AdoMet, an allosteric activator), and heme, the function of which has been subject to intensive debate for many decades (see below for additional details). As a PLP-dependent enzyme, CBS belongs to the β family (or fold type II family) sharing high similarity of its catalytic core with tryptophan synthase β subunit, a prototype of the family [14], responsible for the β-replacement or β-elimination reactions. In the folded protein, this active site can be reached through a narrow channel, the catalytic center of a monomer being structured by two central β-sheets surrounded by α-helices, in between N- and C-terminal domains [15]. 

One of the features that distinguishes CBS from the other PLP-dependent enzymes is its N-terminus containing a heme-binding site. Residues Cys52 and His65 are responsible for coordinating axially the heme in a hydrophobic pocket displayed at the surface of the protein [15,16]. Despite this essential difference with the catalytic site in terms of exposure, the distance between the heme and PLP is approximately only 20 Å [17]. As for the role of the heme, its function remains vague since it is not directly involved in the catalysis, but still influences folding and is sensitive to the redox status of its environment. In addition, recent studies suggest that the first 40 residues of the human CBS N-terminus constitute an intrinsically disordered region, which transiently binds heme via a second binding site, the CP-based motif with Cys15 and His22 as axial ligands [18,19]. While the function of this additional heme-binding site is not fully understood, according to one publication, the CBS Cys15Ser mutant is unable to bind heme at this second binding site and is ~30% less active compared to the WT variant [18]. However, a previous characterization of the CBS Cys15Ser variant showed no effect on enzymatic activity, but rather significant reduction in protein aggregation mediated by formation of intermolecular disulfide bridges [20]. Intriguingly, CBS also contains a CXXC oxidoreductase motif, but several studies report that redox sensitivity is maintained when CXXC motif is mutated while it is lost when heme domain is mutated [21]. The full-length CBS has a C-terminal regulatory domain with a tandem of CBS domains, CBS1 and CBS2, which associate in dimeric assembly to form a Bateman module [22]. Each CBS domain comprises of a three-stranded β-sheet and two α-helices, and together they play an autoinhibitory role by blocking the active site. SAM acts as an allosteric activator of CBS by binding into the cleft within Bateman module followed by domain rearrangement and release of intrasteric block from the catalytic site. In addition to the regulatory role, the C-terminal domain is also involved in the formation of the CBS homotetramer. This conclusion is supported by the observation that the truncated CBS, which lacks the entire C-terminal regulatory domain (about 140 residues), forms dimers. Similarly, CBS dimers are also formed when just 10 residues from the CBS2 domain of the regulatory domain are removed, which facilitated successful crystallization of a full-length human CBS [12,16,17,22].

### 1.3. Regulation of CBS Expression 

The *CBS* gene is located on human chromosome 21 in the subtelomeric region q.22.3 [12] and its entire sequencing revealed 23 exons in 1998 [13], with 15 of them coding for the CBS polypeptide. The two promoters −1a and −1b are found to be mainly used. They are rich in GC and their regions contain numerous putative binding sites for transcription factors but no TATA box, as well as an estrogen receptor binding site. Some of those possible bindings have been confirmed to regulate *CBS* basal transcription, such as specific protein Sp1 and Sp3, upstream stimulatory factor 1 (USF-1), nuclear factor (NF) −Y on −1b promoter [23]. Notably, evidence has been presented that Sp1/Sp3 transactivates the -1b promoter, the increased ratio correlating with increased gene transcription in general (mostly Sp3 can repress transcription driven by Sp1) [24]. The transcription factor NF-E2 p45-related factor-2 (Nrf2) was also shown to induce the *CBS* gene, when stabilized by H_2_S which inhibits Nrf2 repressor Kelch-like ECH-associated protein-1 (Keap1) [25]. Besides, this regulatory mechanism has been reported to be induced by the onion-derived metabolite, S-1-propenylmercaptocysteine [26]. The common pathogenic c.833T>C(p.Ile278Thr) mutation observed in CBS deficiency is often found in combination with 68-bp insertion (844_845ins68), which is an exact duplication of the intron-exon boundary of exon 8 [27]. Interestingly, this variant can be skipped by alternative splicing, leading to the formation of normal mRNA and enzyme activity, but the yield of its transcription is considerably reduced, indicating that proper regulation of *CBS* may depend on this region. 

CBS is a cell- or tissue-specific constitutively expressed enzyme; its expression (and, consequently, its activity) is primarily regulated by post-transcriptional modifications under normal physiological conditions (see below). However, modifications in CBS mRNA (and consequently CBS protein) expression are observed in certain physiological changes and pathophysiological states. Therefore, at the image of its diverse distribution and large potential regulators list (including H_2_S itself), CBS regulation is extremely complex.

#### 1.3.1. Physiological Factors Regulating CBS 

During a microarray experiment with MC3T3-E1 murine pre-osteoblast cells, treatment with the active form of vitamin D, 1,25-dihydroxyvitamin D3 (1,25(OH)_2_D3), showed a fast and strong induction of *CBS* gene transcription [28]. This activation depends on the vitamin D receptor, which binds together with retinoid X receptor and acetylated histone H4 to the vitamin D responsive element in the second *CBS* intron. 1,25(OH)_2_D3 also induced CBS expression in other murine cell lines from bone marrow, mammary carcinoma or kidney (but not in hepatocytes), suggesting that this regulation process is specific for cells and tissues that express sufficient vitamin D receptor. In uterine artery endothelial cells, CBS mRNA and protein levels increase upon treatment with estriadiol-17β, as well as with specific agonists of estrogen receptor (ER) α or β, while their antagonists strongly attenuate CBS up-regulation by estradiol-17β [29]. In accordance with those observations, CBS is up-regulated during pregnancy in uterine artery endothelium and smooth muscle cells compared to the menstrual cycle [30]. The activation of *CBS* promoter by estradiol-17β was also confirmed in vitro, although binding to the ER binding site cited previously remains to be verified. Testosterone has also been proposed as a CBS regulator, since in female Balb/c kidney and castrated male, CBS protein levels are lower than in male kidney [31]. 

Glucocorticoids have also been reported to regulate CBS expression, although the published data are conflicting. In H4IIE cells (a rat hepatoma cell line), glucocorticoids were found to increase the cellular levels of CBS mRNA and protein (moreover, the presence of insulin was found to counteract this stimulatory effect) [32]. In contrast, in an in vivo study, psychological stress (presumably through an increase in circulating corticosterone levels) was reported to be associated with a down-regulation of CBS, most likely through a regulation of Sp3 in the CBS promoter [33]. 

Another type of binding that has been demonstrated is the one of hypoxia-inducible factors (HIF) α and HIF β to a hypoxia-response element (HRE) in the human *CBS* gene [34]. This interaction appears to trigger CBS transcription in glia-derived U87-MG cells in response to hypoxia. However, CBS expression was not induced by hypoxia in human aortic or lung microvascular endothelial cells, suggesting again a cell-type-restricted regulation process. In the rat kidney, CBS expression is reduced after being subjected to ischemia/reperfusion, together with an increase of Sp1 phosphorylation, implying its regulatory role in CBS expression [35]. Alternatively, evidence has been presented that serotonin and dopamine convey resistance to hypothermic cell death [36]. Pharmacological inhibition of CBS activity or siRNA-mediated silencing of CBS both reverse the increase in cell survival observed when rat smooth muscle cells are treated with dopamine or serotonin before hypothermia. In liver, lung, kidney, and heart tissues, CBS proteins levels decrease in response to hypothermia, while pretreatment with serotonin increases CBS expression. CBS was suggested to be up-regulated (and consequently H_2_S production) by serotonin and dopamine via an unknown mechanism [36]. 

Importantly, cell growth and cell proliferation itself can induce the upregulation of CBS, which can lead to discrepancies between culture cell models and in vivo models. Indeed, it has been shown that serum and basic fibroblasts growth factor induce *CBS* transcription via the -1b promoter, in opposition to the down-regulation by contact inhibition, serum-starvation, nutrient depletion, differentiation induction [37]. Lymphocytes and activated T cells were also found to express higher CBS mRNA levels than resting T cells [38]. 

In agreement with its growth-related regulation, CBS expression shows striking changes during development. In the mouse cerebellum, CBS protein levels are low in the prenatal period, but drastically increase during the first three weeks after birth, while in other parts of the brain the increase starts in the late embryonic period and is followed by a decrease during the maturation period [39]. *Cbs* gene expression strongly increases during murine pancreatic development: CBS mRNA levels being about 10 times higher at embryonic stage E15.5 and almost 70 times higher at E17.5 compared to E12.5 [40]. However, the expression in adult islets is similar to E15.5. 

On the other hand, CBS expression is also changing in mouse brain during aging, but this response is shows significant regional differences. In retrospinal cortex layers, CBS protein levels were very similar at 4, 24, and 28 months of age, while in the molecular and granular layer of the Dentate Gyrus CBS expression decreases between 4 and 24 months and increases between 24 and 28 months to reach higher levels than at 4 months of age [41]. To a lesser extent the pattern is similar in the lateral posterior thalamus, and it continuously increases with age in the medial habenular nuclei. Overall, CBS expression is at its highest level in brain of 28-month-old mouse, possibly through a common mechanism of selective protein expression linked to aging and redox imbalance. 

Interestingly, caloric restriction has also been reported to up-regulate CBS expression in various tissues [42,43]. 

#### 1.3.2. CBS Regulation by Exogenous Factors

Xenobiotic agents can also regulate the expression of CBS. In normal human keratinocytes, sub-cytotoxic formaldehyde exposure upregulates CBS several hours after the up-regulation of pro-inflammatory genes [44]. This effect is inhibited by CBS metabolite L-cystathionine, implying a negative feedback role of CBS in the inflammation response. Among pro-inflammatory cytokines, interferon γ and IL-4 also up-regulated *CBS* gene transcription in normal human keratinocytes. Conversely, evidence has been presented that mouse microglia is polarized toward M1 type - producing pro-inflammatory mediators such as IL-1β, IL-6, TNFα and expressing inducible nitric oxide synthase (iNOS) - while exposed to the environmental toxin rotenone, after CBS down-regulation [45]. The mechanism behind this specific CBS regulation remains unclear, although the excess of reactive oxygen species ROS following rotenone exposure may play a role. In rat kidney proximal cells (NRK-52E), uranium exposure leads to the decrease of Nrf2 protein expression and nuclear translocation and down-regulates both CBS and CSE expression; it has been proposed that the suppression of H_2_S production contributes to the cytotoxic effect of uranium in various cells and tissues [46]. In clonal rat pheochromocytoma PC12 cells, the excitatory neurotransmitter glutamate induces excessive activation of NOS and overproduction of NO [47]. At the same time, CBS expression is down-regulated, which is reversed by treatment with the NOS inhibitor asymmetric dimethylarginine (ADMA) [47]. Treatment with nitric oxide (NO) donor S-nitroso-N-acetylpenicillamine (SNAP) decreases CBS protein levels, implying all in all that glutamate may regulate CBS expression via NO [47]. In addition, the active metabolite of the environmental neurotoxin 1-methyl-4-phenyl-1,2,3,6-tetrahydropyridine (MPTP) also inhibits CBS expression in PC12 cells [48]. Another environmental factor that has been found to regulate CBS expression is the exposure to radiation. Indeed, CBS was shown to be up-regulated in human hepatoma HepG2 cells upon irradiation in a dose-dependent manner [49].

Interestingly, CBS has also been reported to be regulated by dietary factors, as well as various therapeutic agents. For instance, tyrosol (a natural phenolic antioxidant phenylethanoid, a component of olive oil) has been shown to up-regulate CBS expression [50]. The anesthetic Zoletil^®^ was reported to up-regulate CBS expression in the brain in vivo [51] while several antipsychotic agents (haloperidol, clozapine, olanzapine and risperidone) were reported to down-regulate it in neuronal cell lines in vitro [52].

### 1.4. Distribution of CBS in Various Cells and Tissues 

In mammals, CBS mRNA and protein are primarily found in the liver, brain, kidney and pancreas [53,54]. It is well known that CBS is abundant in hepatocytes; however, it is also detected at lower levels in hepatic stellate cells and Kupffer cells [55,56]. In the brain, all regions express CBS in various amounts but hippocampus, cerebellum and cerebral cortex seem to have the highest expression [57]. Initially, CBS was localized specifically in astrocytes scattered throughout the six cortical layers, the hippocampal dentate gyrus, the Purkinje layer, the corpus callosum and the olfactory bulb, and in cerebellar Bergmann glia [39]. Subsequently, its colocalization with neuronal markers in the brain cortex was also shown [58], as well as its expression in Purkinje cells and hippocampal neurons [59]. Importantly, CBS is expressed in neuronal stem cells, where it appears to regulate their proliferation and differentiation [60]. Regarding its distribution in the kidney, CBS was identified in glomeruli, epithelium from the proximal tubule and renal collecting duct, and renal interlobular arterial endothelium [61,62]. In the pancreas, CBS was detected both in islet cells and in exocrine cells, in particular in acinar cells [63,64]. 

Other tissues express lower levels of CBS, such as endocrine tissues, the gastrointestinal tract, lungs, the bladder, muscle tissues, adipose tissue and lymphoid tissue [65]. In the heart, CBS was found in the cardiomyocytes, in the coronary artery and in the perivascular adipose tissue [66,67], while in lung CBS was detected in the epithelial cells of the alveoli, in the bronchioles and trachea, as well as in the endothelial and smooth muscle cells of the pulmonary artery [68,69,70,71]. Adrenocortical cells express CBS in adrenal glands [72], while CBS expression in thyroid gland is low but markedly increases in thyroid carcinoma [73] (see also below). 

Regarding the digestive system, CBS is present in the gastric mucosa, colonic epithelium, small intestine, precisely in the jejunum and ileum [74,75,76,77]. CBS can also be found in the spleen [78], in particular in activated T cells [38]. The presence of CBS is relatively important in both male and female reproductive systems, with a noteworthy expression in ovary and intrauterine tissue, and to a lesser extent in the prostate and in the testis [65]. Follicular cells express CBS, although it is absent in oocytes [79], and smooth muscle cells of myometrium and lining blood vessel stain positive for CBS [80]. The placenta, the amnion and the chorion also express CBS [81]. Usually CBS cannot be detected in normal breast tissues, but it is strongly overexpressed in breast cancer cells [82]. Prostatic epithelium, but not the adjacent stroma cells, express CBS [83], along with the bladder, urethra tissues and testis [84,85].

### 1.5. Subcellular Distribution and Translocation of CBS

Under physiological conditions, CBS primarily is a cytosolic enzyme, although in some tissues (e.g. rat liver), a detectable amount of CBS can also be localized in the mitochondrial fraction [86,87]. In conjunction with the pathophysiological upregulation of CBS in various disease conditions (e.g., in colon cancer or in Down syndrome; see below), the increase in total cellular CBS protein level is also associated with an increase in the mitochondrial CBS content [88,89,90].

In response to hypoxia or ischemia, elevated levels of CBS protein have been found in mitochondria, which was found to be the consequence of the regulation of mitochondrial CBS stability by Lon proteases [86] (see also below). 

The regulation of mitochondrial CBS levels and the process by which CBS (which, as with the vast majority of mitochondrial proteins, is synthesized in the cytoplasm on the ribosomes and is subsequently transported into the mitochondria) is incompletely understood. Co-immunoprecipitation of CBS with mitochondrial heat shock protein 70 (mtHsp70) may suggest the potential role of mtHsp70 in regulating its mitochondrial levels and/or activity [86]. Since mutants lacking both CBS1 and CBS2 domains or only CBS2 could not be detected in mitochondria but mutants with only CBS1 are, it is plausible that mtHsp70 interacts with CBS via the CBS1 domain [86]. 

Mutant CBS proteins (which result in a rare metabolic disease, homocystinuria, see below) may exhibit a different intracellular distribution than normal CBS. Casique and colleagues found that misfolded CBS mutants exhibited a punctate appereance, presumably localized in inclusion bodies, compared to the homogenous distribution of wild-type CBS [91]. 

CBS may also enter the nucleus. CBS protein was, indeed detected in isolated nuclei derived from mouse brain or liver extracts and was even localized to the nuclear scaffold [92]. In human liver cancer cells HepG2 and murine liver cells, CBS was found in the nucleus and the cytoplasm [92]. Moreover, SUMOylated CBS has been detected in HepG2 nucleus, suggesting that the post-translational modification regulates its nuclear localization [92] (see also below). Although the functional role of CBS SUMOylation and nuclear transport remains unclear, it has been hypothesized that it is a strategy under high local glutathione demand (for example in early phases of cell proliferation [93]) to ensure cysteine delivery in the nucleus [94]. A recent study from public database confirmed that among 115 enzymes involved in the homocysteine-methionine cycle, CBS was the only one identified in both the cytosol and the nucleus [95].

There is also some evidence for the presence of extracellular (i.e., circulating) “free” CBS in the plasma. The CBS enzyme is most likely the result of release from hepatocytes, especially when the hepatocytes are dysfunctional (injured, or necrotic) and their membrane integrity is diminished. This circulating CBS has even been proposed to be useful as a diagnostic marker to identify subgroups of CBS-deficient patients with distinct genotypes [96,97].

### 1.6. Physiological Roles of CBS 

Determination of the physiological role of CBS is not entirely straightforward for at least two reasons. Firstly, CBS-deficient animal models develop a severe phenotype (including, in some instances, neonatal mortality of CBS^−/−^ mice). Thus, some of the phenotypic alterations observed in these animals are the consequence of developmental problems related to CBS (as opposed to the actual physiological roles of the enzyme in a developed, adult organism). Secondly, pharmacological inhibitors of CBS (see below) have limitations in terms of selectivity and specificity as well as—in many cases—limited cell and tissue uptake. Pharmacological effects of “CBS inhibitors” must be interpreted with caution. Nevertheless, one can make various conclusions based on the combination of biochemical properties of CBS (e.g., CBS enzymology, see below); the functional effect of inactivating CBS mutations in animals and humans (see also below), and the effects of various pharmacological inhibitors or CBS silencing or CBS overexpression studies (in cell-based experiments or in animal studies). However, even when the involvement of CBS in a given biological process is undisputable, it is often difficult to determine if the observed biological effects related to CBS are, in fact, due to upstream alterations (e.g., homocysteine accumulation due to CBS inhibition), downstream alterations (e.g., lack of production of cytoprotective cystathione or H_2_S after CBS inhibition) or global cellular changes (e.g., alterations in cellular glutathione levels and compensatory changes in redox balance).

According to the “classical” CBS concept, the primary physiological role of CBS is the degradation of homocysteine and production of cysteine from essential amino acid methionine (Figure 1). This role is supported by the biochemical data (since homocysteine is a main substrate of the enzyme), animal data (CBS-deficient animals develop hyperhomocysteinemia [98,99,100,101,102,103,104,105,106,107,108]) and clinical data (inactivating CBS mutations result in classical homocystinuria, a rare inborn error of sulfur amino acid metabolism; see below).

The most characteristic feature of CBS-deficient mice is a severe degree of hyperhomocysteinemia (an increase in plasma homocysteine concentration from the physiological 5 µM to approximately 500 µM). Some of the characteristic phenotypical changes in these animals include liver steatosis, facial alopecia, loss of visceral fat and decreased bone mineralization [98,99,100,101,102,103,104,105,106,107,108]. As expected, placing these animals on methionine-deficient diet (to reduce homocysteine formation) improves the condition of these mice, and so does CBS enzyme replacement therapy [109]. The incidence of mortality (or lack thereof) appears to be dependent on the genetic background of the mice [102]. Moreover, the mortality is either dependent on a complete absence of CBS (or possibly may be in part dependent on some structural/scaffolding roles of the enzyme), because engineering of a low-activity mutant form of the enzyme rescues the animals from CBS deficiency-associated neonatal mortality, even though these animals continue to exhibit high circulating homocysteine levels [101]. 

The molecular mechanism of CBS deficiency-associated alopecia is unclear. The mechanism of CBS deficiency-associated liver steatosis and liver dysfunction may be related to the accumulation of hepatotoxic homocysteine and/or the lack of cytoprotective cystathionine and H_2_S generation in the liver, but additional mechanisms (e.g., a dysregulation of thiolase, a key enzyme in beta-oxidation of fatty acids [110] as well as dysregulation of various ATP-binding cassette transporters and nuclear hormone receptors involved in liver lipid homeostasis [111]) have also been implicated. Likewise, the disturbances in bone mineralization seen in the CBS^−/−^ mice may be related to either the homocysteine accumulation or reduced H_2_S biosynthesis, since both homocysteine and H_2_S has been shown to regulate bone mineralization through influencing a variety of factors involved including osteoblast and osteoclast activity and vascular function [112,113,114]. Moreover, disturbances in fat handling seen in the CBS^−/−^ mice (including their significant degree of fat loss [110]) may be related to either the homocysteine accumulation or reduced H_2_S biosynthesis, since both homocysteine and CBS-derived H_2_S has been shown to regulate adipogenesis [115].

CBS^−/−^ mice develop a significant cardiovascular dysfunction. These mice exhibit progressive endothelial dysfunction (i.e., attenuated vascular relaxant responses to acetylcholine and other endothelium-dependent relaxant agents, impaired blood–brain barrier integrity, significant vascular remodeling, increased wall thickness, elevated blood pressure, increased extracellular matrix fiber deposition, and fragmented elastic fibers [109,116,117,118,119,120,121,122,123]) as well as a propensity for thrombosis and atherosclerosis [113,124,125]. These alterations were initially attributed solely to the elevated circulating concentrations of homocysteine, a well-established independent cardiovascular risk factor. According to these early concepts, the increased homocysteine produces disturbances in vascular redox status, diminishes intracellular glutathione levels, reduces nitric oxide bioavailability, and the endothelial dysfunction, in turn, produces secondary pathophysiological alterations, e.g., vascular remodeling, hypertension and propensity to develop atherosclerosis [126,127]. However, more recent data indicate that the lack of CBS-derived H_2_S production may also play a significant role; in fact, the cardiovascular alterations observed in CBS^−/−^ systems have been shown to be prevented or reversed by administration of H_2_S donors [128,129]. These reversal-experiments must be interpreted with caution, because they might reflect a simple functional antagonism: indeed, the deleterious vascular effects of authentic homocysteine (i.e., in the absence of changes in endogenous CBS expression or activity) can also be significantly attenuated by H_2_S donors [130,131,132,133,134,135,136]. Overall, the findings above are consistent with the significant expression of CBS in endothelial cells and the ability of these cells to generate biologically relevant amounts of H_2_S to regulate endothelial and vascular function. One should, nevertheless, point out that endothelial cells also contain the other two H_2_S-producing enzymes (CSE and 3-MST) as well, and H_2_S produced by these other enzymes also regulates a variety of endothelial (and vascular) functions [137,138].

As already illustrated by experiments discussed in the previous paragraph, over the last decade, a novel concept emerged stating that CBS has independent roles not only as a homocysteine-metabolizing enzyme, but also as an enzyme that produces H_2_S. H_2_S is generally viewed as an endogenous vasodilator that regulates vascular blood flow and blood pressure as well as physiological angiogenesis (on its own, and in close cooperation with another endogenous gaseous regulator, nitric oxide) [137,138]. CBS is one of the three major mammalian H_2_S-producing enzymes. Thus, one would predict that CBS-deficient mice (and patients with inactivating CBS mutations) and/or animals treated with pharmacological CBS inhibitors would exhibit lower circulating H_2_S levels, impaired vasodilation, impaired angiogenesis and perhaps a moderately elevated blood pressure (all due to the absence of H_2_S). Moreover, one could also expect that activation of CBS (e.g., through application of the allosteric activator, SAM) would increase H_2_S production and regulate various cardiovascular functions. Surprisingly—even though CBS-deficient mice were available for several decades, and the field of H_2_S biology is about two decades old as well—we only have partial answer to the above questions. There are several studies investigating the differential distribution of CBS in various cells and tissues, and accordingly, CBS-dependent H_2_S production in various cells and tissues is also heterogeneous. Based on the effects of the small-molecule PLP-dependent enzyme inhibitor aminooxyacetatic acid (AOAA; see below), the role of CBS-derived H_2_S was reported to be more important in the liver than in the aorta or the gut [139] (however, AOAA has severe limitations as a “CBS inhibitor” and therefore these findings must be interpreted with caution; see below). Banerjee and colleagues have quantified H_2_S production in murine liver, kidney, and brain tissue and have suggested a significant role for CBS in the process, with CSE also contributing in the liver (in a manner that is dependent on the intracellular concentrations of the enzyme’s substrates) [53]. H_2_S production by liver homogenates from CBS^−/−^ mice is markedly lower than the corresponding H_2_S production by liver homogenates from wild-type controls (when cysteine and homocysteine are used as substrates) [140]. All these data support the conclusion that CBS is a significant source of biologically relevant amounts of H_2_S under physiological conditions. A comprehensive comparison of tissue H_2_S generation between wild-type and CBS^−/−^ (or CBS^+/−^) mice yet remains to be conducted.

Plasma H_2_S measurements also support the view that CBS is a significant source of H_2_S biogenesis in mammals: Jensen demonstrated that circulating H_2_S levels in CBS^−/−^ mice are approximately 30% and 46% lower than corresponding levels in wild-type female and male mice, respectively [141]. The underlying potential gender dependence in CBS regulation has not been comprehensively explored, but the fact that male CBS^−/−^ mice exhibit approximately 3× higher circulating homocysteine levels than controls, while the corresponding increase is only approximately 2× in female mice [141] suggests that the basal CBS expression/activity (and, possibly, physiological importance of CBS in H_2_S generation) is higher in male mice than female mice. Data presented in the same study also showed that circulating H_2_S levels could be doubled by treating the mice with ethionine (2-amino-4-(ethylthio) butyric acid, a methionine analog), which is converted to S-adenosyl-ethionine in vivo, which, in turn, activates CBS in a fashion similar to the effect of SAM); CBS activity in liver from these mice increased even more drastically (approximately 4-fold) [141]. These data indicate that under physiological conditions, H_2_S production from CBS is not maximal and can be further enhanced by allosteric activation of the enzyme. The conclusion that CBS-derived H_2_S plays a physiological role in maintaining (i.e., physiologically lowering blood pressure) is indirectly supported by the above data, as well as by the findings demonstrating that CBS-deficient mice exhibit elevated blood pressure [126,127,142], by data showing that pharmacological inhibition of CBS—alone (and especially in combination with inhibition of CSE)—can elevate blood pressure in rats [143,144]. Moreover, according to a meta-analysis, the c.833T>C(p.Ile278Thr) polymorphism (a 68 bp insertion at 844 in the exon 8, which produces a form of CBS that has lower specific activity and produces mild hyperhomocysteinemia) is associated with a significantly higher risk of stroke [145].

Several reports suggest that central H_2_S production, generated by CBS, by acting in the rostral ventrolateral medulla and potential other central nervous system (CNS) structures, may also be involved in the regulation of blood pressure in health and disease [146,147,148,149]. However, most of these studies rely solely on inhibitors of questionable selectivity (e.g., AOAA, see below) and therefore should be interpreted with caution. 

In addition to the role of CBS in the regulation of vascular function and blood pressure (see above), there are also published data indicating that CBS (or CBS-derived H_2_S) may regulate angiogenesis. In CBS^−/−^ mice angiogenesis [129,150], vascular development [151,152], as well as post-ischemic angiogenesis and reendothelialization [125,153] are impaired, consistently with the known role of H_2_S in stimulating angiogenesis. 

H_2_S is an important regulator of various CNS functions. It is generally accepted that under normal conditions, H_2_S in the CNS acts as a neurotransmitter, neuromodulator, and/or neuroprotective factor. One of the major H_2_S-producing enzymes expressed in the CNS is CBS. Indeed, Abe and Kimura already in 1996 demonstrated that brain homogenates produce significant amounts of H_2_S in a regulated manner: H_2_S production in the brain homogenates could be increased by the allosteric CBS activator SAM and reduced by AOAA. However, the CSE inhibitor PAG only had minimal effects, suggesting that CSE-derived H_2_S production plays a relatively minor role [57]. A subsequent study published in 2002 which—using hippocampal slices from wild-type and CBS^−/−^ mice—implicated CBS-derived H_2_S generation in long-term potentiation, and which suggested that glutamate and electrical stimulation induces H_2_S production in neurons [154] has been subsequently retracted for methodological problems [155]. In the subsequent 20 years, unfortunately, no comprehensive follow-up studies appeared attempting to directly re-evaluate the potential role of CBS (or CBS-derived H_2_S) in the regulation of various fundamental CNS functions. Nevertheless, based on the detailed analysis of the brains of CBS^−/−^ mice, it appears that CBS in the CNS regulates neurodevelopment, especially in the cerebellum [39]. The current understanding regarding the physiological role of H_2_S in the regulation of CNS functions is that H_2_S, generated mainly by CBS in astrocytes and 3-MST in neurons participates in cognition, memory, regulation of the cardiopulmonary functions and neuroprotection [156,157,158], although it should be pointed out that many of these conclusions were obtained by investigating the neuronal effects of chemically generated H_2_S (as opposed to exploring the neuronal effect of CBS overexpression/activation), and/or used “CBS inhibitors” of limited utility such as hydroxylamine or AOAA [158,159,160,161,162]. There are also several reports showing that the expression of CBS in the brain is regulated by various disease conditions. For example, kainate-induced seizures cause an up-regulation of CBS in the CNS of mice [39], in the brain of patients with schizophrenia [163] and so does Down syndrome (see below). However, in other CNS diseases (e.g., Alzheimer’s disease and Parkinson’s disease), CBS expression and H_2_S levels in the CNS are significantly decreased [39,164]. 

As mentioned earlier, besides the brain, the liver is another organ that exhibits high expression of CBS. As discussed above, CBS-deficient mice develop liver dysfunction, most likely due to a combination of the accumulation of a cytotoxic mediator and the deficiency of a cytoprotective mediator (homocysteine and H_2_S, respectively). There are several important physiological regulatory functions of CBS in the liver. As overviewed by Wang and colleagues [165], CBS (and its product, H_2_S) in the liver appears to regulate physiological glucose metabolism, insulin sensitivity, and the biosynthesis of lipoproteins. One of the key aspects of CBS in this context is that its presence confers an antioxidant effect [100,166], presumably—at least in part—related to H_2_S biosynthesis and may be the consequence of several genes in the hepatocytes (Fsp27, Cd36, Syt1, Scd1, and Hsd3b5) that regulate, among others, liver steatosis [167]. Additional factors contributing to the pathogenesis of liver dysfunction in CBS-deficient mice may include disturbances in arginine methylation (which, in turn, can disrupt protein-protein interactions) [168] and the down-regulation of DYRK1A, a serine/threonine kinase and antiapoptotic factor [169]. The interrelationship between the above discussed pathophysiological alterations remains to be further investigated. Although the underlying mechanisms are incompletely understood, it is clear that dysregulation of the liver CBS/H_2_S homeostasis significantly contributes to the pathogenesis of liver fibrosis and liver cirrhosis [64,170].

As discussed in the subsequent chapter, CBS overexpression and H_2_S overproduction is now viewed as an important factor in the bioenergetic activation and metabolic reprogramming of cancer cells. However, there are also some data that indicate that CBS may be important in the regulation of physiological bioenergetic functions. Skeletal muscle ATP levels were reported to be lower in CBS^+/−^ mice than corresponding wild-type controls and this was associated with a reduced exercise capacity and skeletal muscle contractility [171]. While the underlying factors may be complex (and may involve, among others, disturbances in skeletal muscle development and vascularization), part of this dysfunction may also involve a direct bioenergetic (mitochondrial) deficit, as it is associated with the dysregulation of several key mitochondrial genes that regulate mitochondrial electron transport (including cytochrome C oxidase subunit IV), mitochondrial transcription, replication and biogenesis [171]. The relative role of CBS-regulated homocysteine, H_2_S and/or other factors in the above alterations has not yet been elucidated.

As discussed in the subsequent chapter, CBS overexpression and H_2_S overproduction is re-emerging as potential causative factors in the pathogenesis of Down syndrome. Among other alterations, the neurocognitive deficit associated with Down syndrome may be linked to toxic overproduction of H_2_S in the CNS. There may be also some evidence, however, that CBS-derived H_2_S may also affect cognition in the general population. For instance, in a rat study investigating the mechanisms of sleep-deprivation-associated cognitive impairment, it was reported that the development of cognitive dysfunction was associated with down-regulation of CBS expression in the CNS; restoration of H_2_S levels (using a chemical H_2_S donor) improved cognitive performance [172]. It is also interesting to note that in a study conducted in a general pediatric population, the c.844_845ins68 CBS allele (a polymorphism of CBS which leads to alternative splicing, but still permits synthesis of normally spliced mRNA) was significantly underrepresented in children with high IQ [173], while the same allele was significantly overrepresented in diabetic patients presenting with mild cognitive impairments [174].

There are also a handful of reports indicating that CBS may be involved in the physiological regulation of immune and inflammatory responses, either as a protective factor (suppressor of the expression of pro-inflammatory mediators) [44,175,176] or in some cases as a pro-inflammatory factor (stimulating the expression of pro-inflammatory mediators) [78] or as a regulator of T-cell activation [38]. While CBS mRNA or protein is not detected in monocytes, the differentiation into macrophages induces CBS expression, concomitant with increasing intracellular levels of SAM, cysteine and glutathione [177]. However, when monocytes are incubated with lipopolysaccharide (LPS), CBS increase is delayed. Interestingly, pharmacological inhibition of CBS in macrophages diminishes *Mycobacterium smegmatis* clearance. In addition, CBS-deficient mice were found to be prone to develop autoimmune disease [178]. However, the published body of data is incomplete; in contrast to the above reports suggesting a beneficial and protective role of CBS in immune responses, in mycobacterium infection model endogenous CBS was actually found to be detrimental and appeared to promote bacterial replication and invasion [179]. Clearly, further work is needed to delineate the role of CBS in particular (or of the various H_2_S-producing enzymes, in general) in the regulation of immune/inflammatory responses.

Although CBS is expressed in various endocrine and exocrine cells and tissues that are important in the regulation of hormone production and endocrine balance, the information related to the potential physiological regulation of endocrine or exocrine hormone secretion is limited. In one study, lentiviral CBS overexpression in the paraventricular nucleus of hypothalamus was reported to increase the expression of pre-TRH expression, elevated plasma thyroxine and thyrotropin level, while decreased the plasma ACTH and corticosterone levels [180]. These effects were associated with lower food intake and decrease body weight and fat mass. These findings may suggest (but certainly do not prove) that physiological, endogenous CBS also plays a role in the regulation of the above systems. There are indirect data suggesting that CBS may regulate insulin secretion [181]. CBS-derived H_2_S has also been implicated in the maintenance of physiological erythropoietin production (and the maintenance of normal erythropoiesis), at least in part through the maintenance of iron homeostasis and the maintenance of expression of various iron-metabolism proteins, including; two key enzymes involved in the heme biosynthetic pathway, delta-aminolevulinate synthase 2 and ferrochelatase [182,183,184,185]. Finally, studies by Wang and colleagues, using both genetic (CBS silencing) and pharmacological (AOAA) approaches indicate that CBS (most likely, via generation of H_2_S) plays a role in the maintenance of adrenocorticotropic hormone-stimulated corticosterone production [72].

### 1.7. Homocystinuria 

A large body of literature (approximately 300 articles) relates to the role of CBS mutations in the pathogenesis of classical homocystinuria, a rare inborn error of sulfur amino acid metabolism caused by the deficiency of CBS activity. Homocystinuria is characterized by a massive accumulation of homocysteine, which, in turn, produces a variety of clinical symptoms. There are various experimental and early-stage clinical approaches that attempt to treat this condition, based either around the reactivation of the dysfunctional CBS protein or enzyme replacement therapy. Since the focus of the present article is CBS inhibition and CBS inhibitors (as opposed to CBS activation or CBS replacement therapy), the reader is referred to extensive expert reviews on the genetic basis, diagnosis, pathogenesis and experimental therapy of homocystinuria [17,166,186,187,188,189,190,191,192,193].

## 2. The Biochemistry of CBS 

### 2.1. Organization of the Active Site of CBS

**Figure 4:**
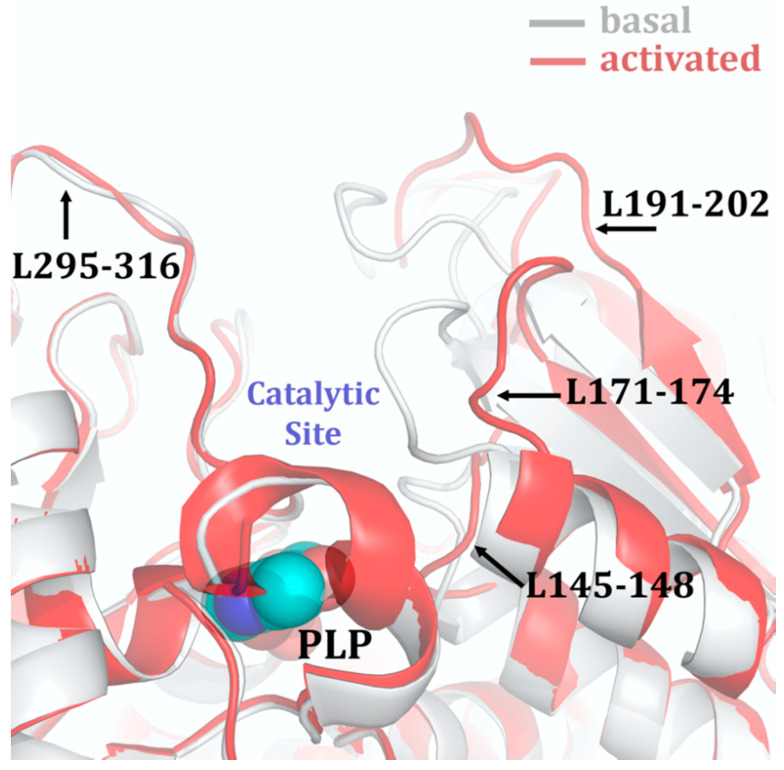
Structural elements determining the access to the active site of hCBS. A zoom-in view showing the structural elements delineating the entrance to the catalytic cavity in the SAM-free basal (grey) and in the SAM-bound activated (red) conformations of hCBSΔ516-525 in the absence of bound substrates. In the activated conformation, the loops L145–148, L171–174, and L191–202 adopt an open conformation that allows free access of substrates, whereas in the basal conformation these loops remain closed and compressed towards the catalytic center by structural elements from the regulatory domain of a complementary subunit (not shown for clarity). Reproduced by permission [195].

The CBS catalytic process and the function of its catalytically active PLP cofactor have been extensively studied in the past. The crystal structure of the truncated human CBS lacking the C-terminal regulatory domain revealed that the PLP cofactor is linked to the -amino group of Lys119 residue via a Schiff base linkage forming an internal aldimine [18,20]. The pyridinium nitrogen and the phenolic oxygen of the PLP cofactor form hydrogen bonds with Ser349 and Asn149 residues, respectively, while phosphate moiety of the PLP is stabilized by an extended hydrogen bonding network with residues of the Gly256-Thr257-Gly258-Gly259-Thr260 loop. Together, these residues anchor the PLP deeply in the protein matrix and the active site is accessible only through a narrow channel. Conformation of the loops delineating the PLP-containing cavity, namely L145-148, L171-174, and L191-202, defines accessibility of the catalytic center by the substrates and thus the activity of the enzyme (Figure 4) [19,194]. 

**Figure 5:**
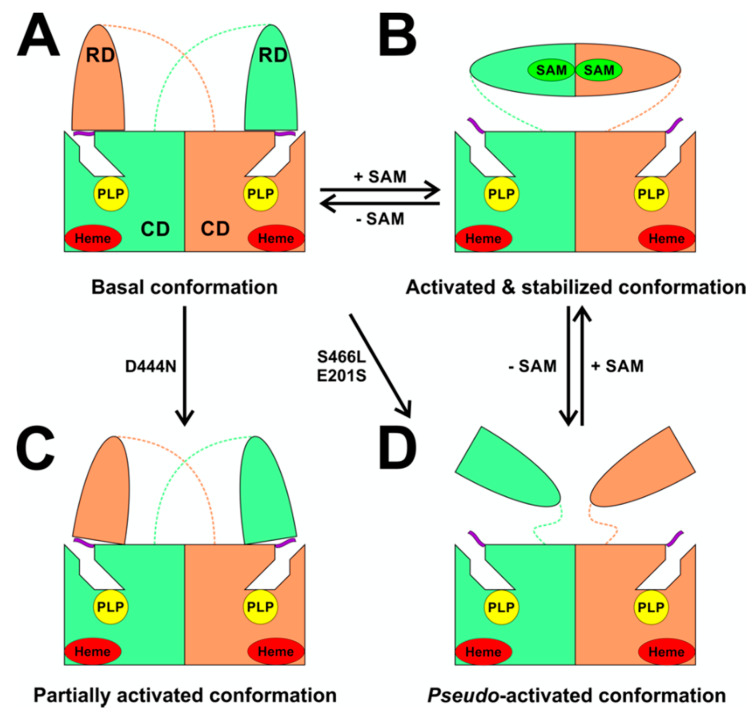
A model of SAM-mediated allosteric regulation of hCBS. (**A**)The engineered hCBSΔ516-525 construct is catalytically indistinguishable from the native hCBS WT, but assembles into dimers (light green and orange subunits) compared to native tetramers. Each subunit contains heme (in red oval) and PLP cofactor (in yellow circle). In the absence of SAM, the enzyme is in the basal conformation with low specific activity (~200 U/mg of protein), where the regulatory domain (RD) of one subunit interacts with the loops delineating entrance to the catalytic cavity of the other subunit (purple ribbon) and thus limits the activity of the complementary catalytic domain (CD). (**B**) Binding of SAM (in green oval) leads to a displacement of the regulatory domain away from the catalytic cavity and formation of a disk-shaped CBS module. Thus, auto-inhibition is released, and the enzyme is activated approximately 5-fold. Activated conformation is stabilized by the presence of SAM in the site S2. (**C**) Pathogenic mutations, such as Asp444Asn, may disrupt interaction between the RD and the CD as well as impair SAM binding thus yielding partially activated conformation. Such structural perturbation enables increased flexibility of the loops near the catalytic cavity and leads to an increased catalytic activity of the mutant enzyme. (**D**) Other mutations, such as the pathogenic Ser466Leu or artificial Glu201Ser, completely abolish interaction between the RD and the CD yielding a pseudo-activated conformation. If SAM binding is not impaired by the mutation, additional presence of SAM may result in the formation of CBS module and thus “true” activated and SAM-stabilized conformation. Reproduced by permission [195].

The loops are in a “closed” (collapsed) conformation when the substrate occupies the catalytic cavity or when the C-terminal regulatory domain sterically interferes and thus limits access to the PLP center. On the other hand, the loops are in an “open” (relaxed) conformation in the absence of the substrate in the PLP cavity or when the steric block imposed by the regulatory domain is relieved by its removal (in the truncated enzyme), binding of SAM or the presence of activating missense mutations, such as artificial Glu201Ser or pathogenic Asp444Asn [195,196] (Figure 5).

The presence of heme in human CBS absorbing at 428 nm limits direct visualization of PLP-based reaction intermediates and therefore much of the spectroscopic characterization of CBS catalytic mechanism was performed on heme-independent yeast CBS [196,197,198,199,200]. CBS catalysis follows a ping-pong mechanism (reviewed in [201]). Briefly, addition of the first substrate serine disrupts the internal aldimine formed between PLP and the Lys119 residue and rapidly leads to a formation of the external aldimine of the PLP with serine. Subsequent deprotonation of the substrate results in a formation of carbanion intermediate, which rapidly converts into aminoacrylate (a stable key reaction intermediate), following β-elimination of water from the external aldimine [194]. The second substrate homocysteine nucleophilically attacks aminoacrylate to yield an external aldimine with cystathionine. The release of the reaction product restores the internal aldimine with the Lys119 residue. Stopped-flow spectroscopic analyses suggested that the conformational change leading to the product release is likely the rate-limiting step of CBS catalysis [198,202], also supported by the crystal structures of fruitfly CBS in the absence and presence of a substrate [194].

Pathogenic missense CBS mutations causing homocystinuria were shown to decrease the affinity of the enzyme to the PLP cofactor causing lower saturation of the enzyme with the PLP, which results in impaired catalytic activity [203]. A study using patient-derived fibroblasts showed that CBS mutants with a moderately reduced affinity for PLP can be rescued by supplementation of pyridoxine (vitamin B6), a precursor of PLP, unlike those mutants with more dramatically reduced affinity for the cofactor. As the molecular mechanism conferring pyridoxine responsiveness remains unknown, particularly due to lack of correlation in data obtained from cellular and animal models of homocystinuria and patients, the potential benefit of pyridoxine supplementation remains to be confirmed empirically. A natural history study of homocystinuric patients suggests that the most prevalent pan-ethnic p.Ile278Thr mutation and other mutations (e.g., Ala114Val and Arg226Lys) confer pyridoxine responsiveness in patients, while, for example, the Irish Gly307Ser and the Spanish Thr191Met mutations appear to be incompatible with pyridoxine responsiveness [204]. Pyridoxine, therefore, may act as a pharmacological chaperone stabilizing the structure by increasing saturation of the mutant enzymes with the PLP, leading to increased steady state levels of CBS protein and ultimately rescuing the CBS activity [188].

### 2.2. H_2_S Biosynthesis and Other CBS-Catalyzed Biochemical Reactions

**Figure 6:**
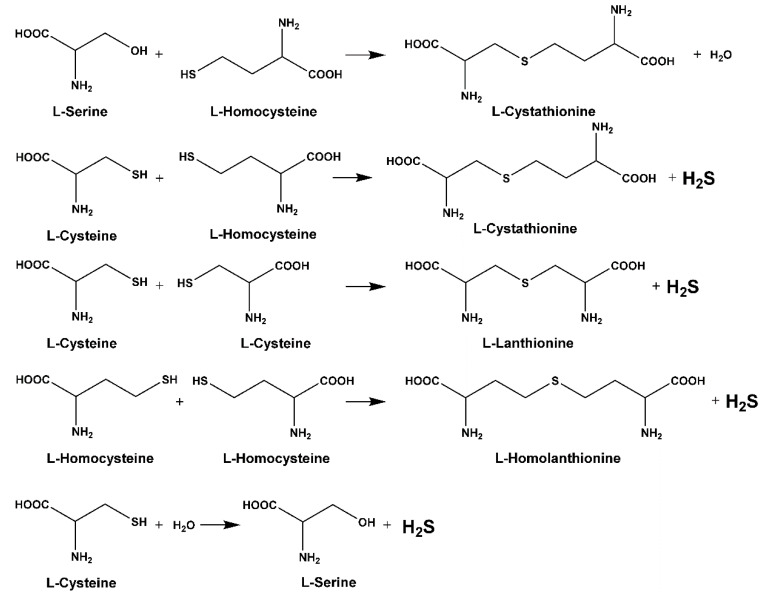
Key biochemical reactions catalyzed by human CBS. Only those reactions are shown, which are physiologically relevant using naturally occurring substrates in the cell. All reactions follow β-replacement/elimination mechanism.

Sequential and structural similarities assign the CBS catalytic core into a β (or fold type II) family of PLP-dependent enzymes [205]. Members of this family catalyze α,β-replacement/elimination reactions, which all follow the catalytic mechanism outlined above. The canonical CBS reaction is a β-replacement of serine with homocysteine forming cystathionine and water. However, with broadly defined reaction specificity and inherent substrate promiscuity, CBS catalyzes several alternative reactions leading to H_2_S production (reviewed in [206,207,208]) (Figure 6). 

Considering only the physiologically relevant substrates, cysteine can substitute for serine, which leads to production of cystathionine and H_2_S in the presence of homocysteine [209]. CBS can form H_2_S by using cysteine either via the β-elimination mechanism yielding serine and H_2_S or via β-replacement with another molecule of cysteine leading to the formation of lanthionine and H_2_S [140,210]. Notably, two thirds of the lanthionine pool come from condensation of serine with cysteine, i.e., the alternative CBS reaction, which does not contribute to H_2_S biogenesis [140]. The most kinetically relevant, alternative H_2_S-generating CBS reaction is the condensation of cysteine with homocysteine; this contributes to over 95% of H_2_S compared to less than 5% when cysteine is used alone [210]. However, the in vitro enzyme kinetics is not favorable for the alternative H_2_S production by CBS compared to the canonical reaction. The specificity constant kcat/km for the canonical serine and homocysteine reaction is 2–5-fold higher than for the alternative condensation of cysteine and homocysteine [140]. The preference of CBS for serine as a substrate is mostly determined by the affinity of CBS for its substrate, which is 7–10-fold higher for serine over to cysteine.

It is not completely understood which factors determine CBS catalysis in vivo. In vitro modeling suggested that the serine to cysteine ratio is the main determinant of CBS-catalyzed biogenesis of H_2_S [140]. Abundance of cysteine in the extracellular compartment, such as plasma, over serine allowed for over 43% of CBS activity leading towards H_2_S biogenesis. On the other hand, excess of serine over cysteine typical for the intracellular compartment limited such alternative reactivity to less than 1.5%. Considering the pathophysiological effects of CBS expression in the regulation of H_2_S homeostasis in cancer or Down syndrome (see below), other factors, such as hypoxia, may influence CBS reactivity. In addition, it is not known if interactions of CBS with either a small-molecule modulator or a protein impact the affinity of CBS for its substrates or its kinetic efficiencies in vivo.

## 3. Physiological Regulation of CBS Enzymatic Activity 

### 3.1. Allosteric Activation of CBS by SAM 

**Figure 7:**
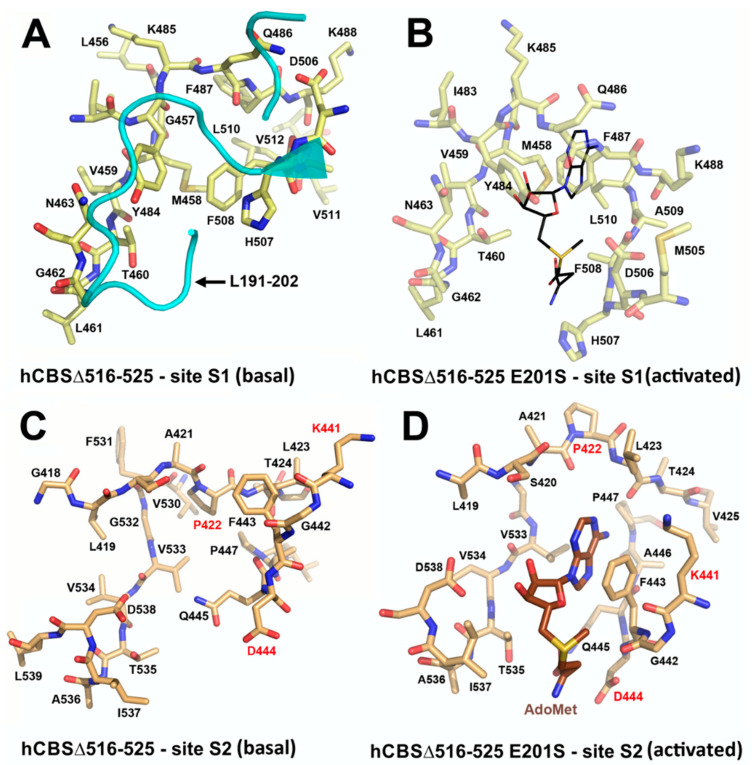
SAM binding sites in the basal and the activated conformation of hCBS. (**A**) Site S1 in basal conformation of hCBSΔ516–525. The entrance to site S1 is sterically blocked by the presence of structural elements from the catalytic core of a complementary monomer in the dimer (cyan). Additionally, bulky hydrophobic residues occupy the cleft and impede with the binding of SAM at this site. (**B**) Site S1 in activated SAM-bound conformation of hCBSΔ516–525 Glu201Ser mutant. Despite the presence of SAM during the crystallization, site S1 remains empty. As shown, binding of SAM (modeled in black lines) would cause steric clashes within the site S1, even in the activated conformation of hCBS. (**C**) Site S2 in basal conformation of hCBSΔ516–525. The site S2 is fully solvent-exposed and is not blocked by bulky residues. (**D**) Site S2 in the activated SAM-bound conformation of hCBSΔ516-525 Glu201Ser mutant. This site represents the only identified SAM-binding cavity in hCBS crystal structure. The site S2 shows a hydrophobic cage that hosts the adenine ring of SAM, conserved Asp538, Thr535 and Ser420 residues stabilizing the ribose ring, and a hydrophobic residue (Ile537) preceding Asp538 that accommodates the alkyl chain of SAM. Note that SAM binding induces a relative rotation of the two CBS motifs that results in a slight reorientation of the residues within the site S2. In the absence of such structural change, accommodation of SAM within the site S2 would be sterically impeded. Reproduced by permission [195].

Among many functions, SAM regulates the flux of organic sulfur through competing transsulfuration and remethylation pathways by allosteric activation of CBS and inhibition of MTHFR (Figure 1) [211]. The regulation by SAM represents the most important, but not completely understood modulatory mechanism of CBS, which goes beyond simple activation of CBS catalytic activity (reviewed in [191]). Calorimetric studies showed that a total binding capacity of human CBS is six SAM molecules per native CBS tetramer with two SAMs binding to high-affinity sites (Kd 10 nM) and four SAMs to low-affinity sites (Kd 400 nM) [212]. SAM first kinetically stabilizes the regulatory domain, as demonstrated by significantly decreased denaturation rates in vitro. Kinetic stabilization of CBS by SAM was previously demonstrated in vivo [213]. Increasing concentrations of SAM further stabilize CBS, but SAM also increases the catalytic turnover of the enzyme. SAM is a V-type activator of CBS meaning that it increases catalytic efficiency by increasing Vmax of CBS without any significant effect on affinity of the substrates (km). Crystal structures of human CBS in both the SAM-free basal and the SAM-bound activated conformations provided further insight into molecular mechanism of the allosteric regulation of CBS by SAM (Figure 7) [19,195,196]. 

In the absence of SAM, the regulatory domain of one subunit in the dimer is placed atop of the entrance to the catalytic cavity of the other subunit pushing the loops delineating the entrance to the catalytic site and thus sterically limiting the flux of substrates and products in and out. The CBS domains CBS1 and CBS2 found in the regulatory domain are well-known to be associated with binding of purine analogs in various proteins. Therefore, each CBS subunit contains two potential SAM bindings sites. However, crystal structures of human CBS showed that only one site can accommodate SAM, while the other site is blocked by several bulky hydrophobic residues (Figure 7) [19,195,196]. Binding of SAM into the only available site induces rotation of the CBS domains, which weakens their interactions with the loops of the catalytic cavity. Subsequently, the CBS domains stabilized by SAM from both subunits of the dimers associate together to form an antiparallel CBS module [195,196]. The CBS module lies on top of the catalytic core with minimal interactions, thus allowing free movement of the loops delineating the catalytic cavity resulting in activation of the enzyme. Such conformation strongly resembles that of fruitfly CBS, which has high catalytic activity similar to SAM-activated human CBS but does not bind SAM [194,214]. The discrepancy between SAM-binding stoichiometry determined by calorimetric versus crystallography techniques was apparently caused by the oligomeric status of the proteins used in the respective studies [215]. Removal of the residues 516-525 from the regulatory domain of human CBS results in the formation of dimers, which facilitated crystallization studies, compared to tetramers of native CBS. Although such change does not impair its activation by SAM, it apparently eliminates two high-affinity sites responsible for kinetic stabilization of the native enzyme. These data suggest that oligomeric status modulates SAM binding and thus may represent an additional mode of CBS regulation.

### 3.2. Post-Translational Modifications of CBS Affecting Its Activity or Expression 

As discussed above, the catalytic activity of CBS is importantly affected by its supramolecular assembly (i.e., tetramerization) as well as by its principal allosteric modulator, SAM. Naturally (as with any other enzyme), the rate of CBS catalysis is also expected to be regulated by its substrate level. Nevertheless, cell-based direct studies are limited in this regard; substrate-based regulation is principally based on in vitro biochemical studies that rely on various assumptions regarding the intracellular levels of CBS substrates. Finally, there are speculations that protein-protein interactions involving CBS may also affect the catalytic activity of this enzyme. In particular, two interactions have been recently discussed [216]: the interaction of inosine-5′-phosphate dehydrogenase through its CBS domain with saglifehrin-bound cyclophilin A (functional response: modulation of cell growth) and the interaction of methionine adenosyltransferase with CBS domain containing chloroplastic-like protein; the latter interaction was demonstrated in wheat in response to stress conditions. The exact relevance of these protein-protein interactions of CBS remains to be further elucidated. A third putative interaction occurs between CBS and Huntingtin protein; this interaction has been proposed in the pathogenesis of excitotoxic neuronal damage [217]. Although this latter interaction has been described over 20 years ago, we were unable to find any follow-up studies investigating its mechanism or its pathophysiological significance.

For a long time, the functional role of heme in CBS remained an enigma. Recent studies by Banerjee and colleagues indicate that the heme in CBS may play an important role in switching the transsulfuration pathway from the generation of cysteine production to the biosynthesis of H_2_S [202]. In this context, it is especially interesting that the heme in CBS is subject to a variety of modifications by various labile biological species. However, CBS heme needs to be first in a reduced ferrous (Fe^2+^) form compared to its natural highly stable oxidized ferric (Fe^3+^) form to function in a ligand binding and regulation of CBS activity. Since CBS heme has a very low redox potential (−350 mV) and ferrous form of CBS is highly unstable and rapidly inactivated in vitro, the physiological feasibility of this CBS regulatory mechanism was an open question. This subject has been comprehensively reviewed recently [218]. In short, the heme (similar to many other heme groups, e.g., the one in soluble guanylate cyclase), binds both NO and (with significantly lower affinity), CO as well. The binding of either of these two species produces an inhibitory effect on CBS. Interestingly, the heme of CBS can also catalyze side-reactions that yield superoxide (from oxygen) or NO and peroxynitrite (from nitrite) [219,220]. The biological significance of these side-reactions is currently unclear.

The most common post-translational protein modification is phosphorylation. In 2008 Ragunathan published the crystal structure of a hypothetical protein ST2348 (PBD ID: 2EF7) from the hyperthermophilic bacterium *S. tokodaii* containing a tandem of two CBS domains and identified the highly conserved residue Asp118, located in a negatively charged patch near the ligand binding cleft and hypothesized that this amino acid could serve as a site for phosphorylation [221]. A subsequent report identified multiple phosphorylation sites of a set of recombinant nucleotide-binding proteins in *E. coli*, including kinases and CBS domain containing protein [222]. With respect to the mammalian CBS, the experimental evidence is limited. In human bladder and urothelial T24 cell lines stimulated with muscarinic receptor agonists, experimental evidence for CBS-cGMP/PKG-dependent phosphorylation of CBS was reported at Ser227, which, in turn, appeared to stimulate the activity of the enzyme, as demonstrated by increased H_2_S generation [223]. Computational studies and phosphoproteome analysis of various normal and transformed cells identifies or predicts further phosphorylation sites of human CBS, most consistently on Ser32 and Ser199 (www.phosphosite.org) [224,225,226,227,228,229,230,231,232,233,234,235,236,237], but the functional role of these putative modifications has not yet been tested experimentally. If (similar to many other enzymes), phosphorylation of CBS confers an activating effect, then theoretically, inhibitors of the kinase(s) involved in this process may serve as an indirect way to suppress the activity of CBS. 

CBS can be S-glutathionylated on Cys346, which, in turn, was found to enhance its activity ∼2-fold in vitro [238]. The S-glutathionylation, and the increase of the catalytic activity of CBS, was further increased under conditions of oxidative stress, as demonstrated in HEK293 cells exposed to hydrogen peroxide [238]. Because H_2_S is known to exert both direct and indirect antioxidant effects (i.e., through reactions with various pro-oxidant species and/or through the up-regulation of various intracellular antioxidant systems, at least in part through Nrf2 activation) these data indicate that S-glutathionylation, and subsequent increase of H_2_S production may serve as a protective or counterregulatory (i.e., antioxidative) mechanism. However, a CBS-mediated antioxidative effect may be (at least in part) counterbalanced by a direct, oxidative-stress-mediated inhibition of the catalytic activity of CBS. Niu and colleagues, using human recombinant CBS enzyme in vitro, and HEK293 cell systems, demonstrated that oxidative stress can reduce the catalytic activity of CBS by 50–70% through the redox modulation of its 272-CXXC-275 motif (i.e., through the modulation of the disulfide/thiol balance) [239]. Taken together, we must conclude that the net effect of increased oxidative stress on CBS activity can either be an increase or a decrease, depending on the experimental or cellular conditions.

Another common form of post-translational modification is the attachment of large covalent tags to acceptor proteins such as SUMO (small ubiquitin-like modifier) or attachment of ubiquitin (i.e., SUMOylation and ubiquitination, respectively). The SUMOylation of CBS was first demonstrated in 2006 [92]; the C-terminal regulatory domain of CBS was found to be obligatory for the SUMOylation process; when SUMOylated, CBS translocated into the nucleus (although the functional role of this translocation has not been determined). SUMOylation inhibited CBS catalytic activity; this inhibition is further exacerbated when the experimental conditions also include human polycomb group protein 2 (hPc2), an interacting partner of CBS that is involved in promoting the SUMOylation reaction [94]. 

In contrast to the available information on SUMOylation, there is only limited information published on CBS ubiquitination, although ubiquitination is a common post-translational modification of cellular proteins (which, in turn, regulates key cellular processes including membrane trafficking and protein degradation). Nevertheless, in 2008, using the UbiSite approach for comprehensive mapping of lysine and N-terminal ubiquitination sites, Akimov and colleagues identified Lys72 and Lys481 of human CBS as two significant ubiquitination sites [240]. Ubiquitination is a common protein ‘tagging’ process, which facilitates the proteosomal degradation of most proteins. It can be involved in the degradation of excess or misfolded proteins, but it is also a key system in regulating physiological protein degradation and turnover [241]. Recent studies by the Kruger group have tested the effect of pharmacological inhibition of proteosomal activity on the intracellular levels and activity of CBS. These experiments were designed in the context of the experimental therapy of inactivating CBS mutations causing homocystinuria (see above) and therefore used experimental systems involving missense mutant human CBS enzymes which have a markedly reduced catalytic activity (CBS variants containing pathogenic missense mutations p.Ile278Thr or p.Ser466Leu) [242]. Treatment with two different proteasome inhibitors (ONX-0912 and bortezomib) increased CBS protein levels as well as catalytic activity [242]. The above data, taken together, indicate (although do not prove) that ubiquitination and subsequent proteosomal degradation is a significant post-translational regulatory pathway not only for mutant CBS, but for the normal, physiological enzyme as well. 

As mentioned earlier (see above), CBS is also subject to degradation (cleavage) by various proteases, with subsequent changes in the activity of the enzyme. The first evidence for such a regulatory mechanism was shown in a report by Skovby, Kraus, and Rosenberg in 1984 who demonstrated that—in addition to the regular, approx. 63 kDa Mw form of CBS, liver extracts also contained a shorter (~48-kDa) CBS protein. This lower-molecular weight form of CBS could be recreated in vitro by trypsin incubation (i.e., limited proteolysis), and this was associated with an increase in the catalytic activity of CBS [243]. A subsequent study by Zou and Banerjee in 2003 also reported a lower-molecular weight CBS (with increased activity compared to the physiological form) in hepatocytes subjected to pro-inflammatory stimulation (TNF-α) in vitro [244]. (In contrast to the cleavage process, the pro-inflammatory cytokine did not up-regulate CBS mRNA or total protein expression). Increased intracellular ROS production and a subsequent proteosomal cleavage process was implicated in the process [244]. The process of CBS cleavage has also been demonstrated in vivo, in the livers of endotoxin-treated mice [244]. One can hypothesize that the truncated CBS version demonstrated in these early studies is identical to the truncated CBS lacking the regulatory domain (45CBS). As discussed earlier, 45CBS is considered the evolutionarily conserved active core and which has a higher specific activity than the physiological form of the enzyme (but is no longer regulated by SAM) [243,245,246,247]. Interestingly, in a recent study, a 45-kDa form of CBS was only detectable in the liver (but not in the brain) of mice [41], indicating that perhaps there is a physiological proteolytic regulation of CBS, but this may well be cell-type and tissue dependent. 

Finally, it should be mentioned that a specific form of proteolytic CBS regulation has recently been identified by Rui Wang and his colleagues. This relates to a particular, mitochondrial form of proteases, called Lon proteases, major protein degradation enzymes located in the mitochondrial matrix. As mentioned earlier (see above), a fraction of CBS is localized to the mitochondria (at least in some—perhaps not all—cells and tissues) under physiological conditions. However, in certain disease states (e.g., certain cancers or in Down syndrome, see above), mitochondrial CBS content increases. Wang and colleagues demonstrated that ischemia (in vitro) or hypoxia (in vivo) increased the accumulation of CBS proteins in mitochondria of hepatocytes, and this response was, at least in part, due to Lon protease activity [86]. According to the mechanism unveiled by the Wang group, Lon protease degrades mitochondrial CBS because it specifically recognizes the oxygenated (but not the deoxygenated) heme of CBS. Ischemia or hypoxia leads to the mitochondrial accumulation of CBS, because ischemia increases the proportion of deoxygenated heme, and this is no longer recognized by the Lon protease [86]. The molecular weights and the specific activities of these cleaved CBS protein fragments remain to be defined, but based on H_2_S measurements [86], they appear to be less active than the mitochondrially localized native CBS.

One of the primary foci of the current article is to outline the various approaches by which CBS can be inhibited. The allosteric activation mechanisms of CBS, as well as the various post-translational modifications offer several indirect approaches to do so. Moreover, there may also be indirect approaches related to decreasing the substrate availability of CBS, for instance by blocking the transport of cystine into the cells. This can be achieved, for example, by blockers of the cystine/glutamate antiporter system Xc- [248,249,250]. 

Indirect approaches to reduce CBS activity (as well as their potential off-target effects) are summarized in Table 1.

## 4. Disease Conditions in Which Inhibition of CBS is Expected to Be Beneficial 

**Figure 8:**
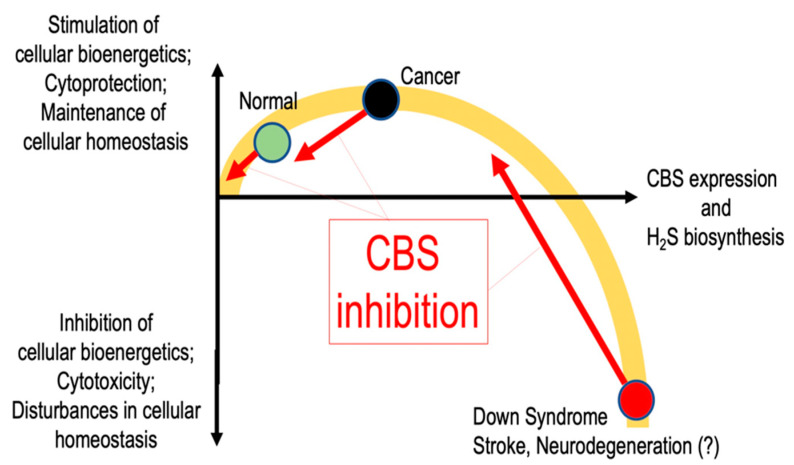
The bell-shaped role of CBS expression and H_2_S biosynthesis in the regulation of cell viability in health and disease. CBS inhibition can impair cancer cell viability by reducing the formation of H_2_S which the cancer cells use as a cytoprotective factor and bioenergetic “fuel”. CBS inhibition can also improve cell viability, for instance in Down syndrome, by normalizing the toxic overproduction of H_2_S.

Dysregulation of CBS and subsequent pathophysiological alterations in cellular H_2_S levels have been implicated in several diseases. For instance, in many cancer cells, CBS up-regulation produces elevated H_2_S levels, which the cancer cells use to drive their accelerated metabolism and proliferation and as a protective mechanism against anticancer therapies and perhaps against elimination by the host immune system. In other conditions (best characterized in Down syndrome), elevated CBS levels yield toxic concentrations of H_2_S, which are deleterious to the cell. The bell-shaped relationship between CBS expression and cell function and cell viability is depicted in Figure 8.

### 4.1. Down Syndrome 

As described above, *CBS* gene is located on human chromosome 21, the chromosome that has an extra copy in trisomy 21 called Down syndrome. Based on the knowledge of homocystinuria and the opposite clinical observations in DS patients, it was hypothesized in 1975 already by Lejeune that an over-activation of the transsulfuration pathway produces an over-use and subsequent decrease in homocysteine levels [251]. 

Ten years later, the up-regulation of CBS enzyme (an expected “gene dosage” effect) and a consequent increase in CBS enzymatic activity was demonstrated in fibroblasts from Down syndrome individuals [252]. This finding strengthened Lejeune’s hypothesis as of CBS possibly contributing to the metabolic imbalance associated with Down syndrome [253]. The up-regulation of CBS in various cells and tissues of individuals with Down syndrome was subsequently confirmed and extended to many cells and tissues—including neurons and brain tissue [90,95,254,255,256,257], as well as in those animal models of Down syndrome which included a triplication of the cbs gene (which, in mouse, is located in chromosome 17) [258,259]. (It should be, nevertheless, noted that the genes located on chromosome 21 are located on 3 different mouse chromosomes, and many of the mouse models of Down syndrome, unfortunately, do not include murine C*BS* [260], and therefore are only of limited translational relevance for the human disease). As expected, CBS up-regulation in Down syndrome resulted in low plasma and tissue homocysteine levels [261,262]. Moreover, in subsequent studies, hundreds of genes were found to be dysregulated in individuals with Down syndrome, the majority of which are not even encoded on chromosome 21 [263,264,265,266,267,268]. These findings underline the complex pathogenesis of Down syndrome and predict that any given single enzyme or biochemical pathway (including CBS or the transsulfuration pathway) can only have a partial role in the pathogenesis of this condition.

**Figure 9:**
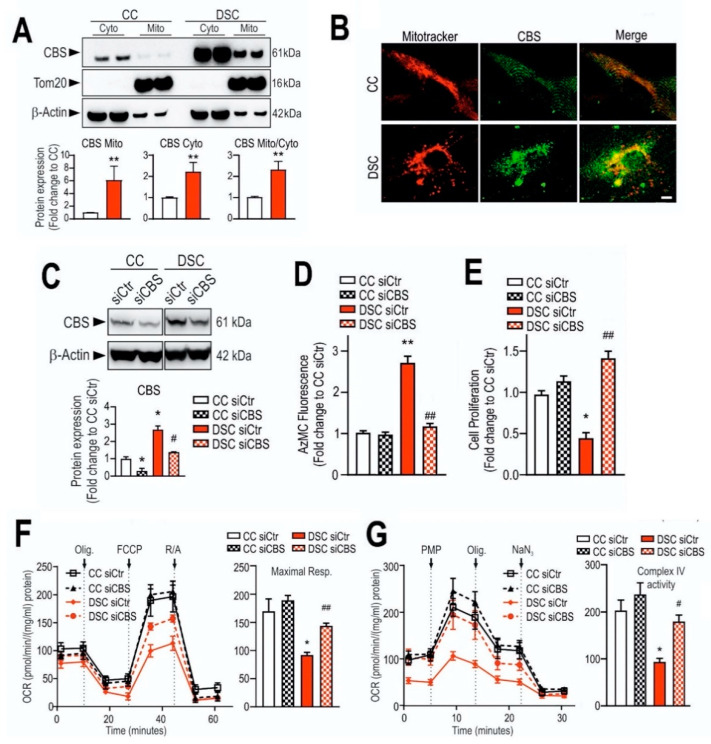
CBS contributes to the suppression of mitochondrial function in Down syndrome fibroblasts. Dermal fibroblasts from a female individual with DS (Detroit 539: DSC) exhibit markedly higher CBS expression, which is, in part, localized to the mitochondria, than cells from an age-matched healthy female subject (control cells, Detroit 551: CC), shown by (**A**) Western blotting and (**B**) confocal microscopy. (**C**) SiRNA-mediated silencing of CBS in DSCs reduces CBS protein expression to a level comparable to the expression seen in CCs, (**D**) reduces the H_2_S overproduction observed in DSCs as opposed to CCs (measured by the fluorescent H_2_S probe AzMC) and (**E**) restores the proliferation of DSCs to values comparable to CCs. In addition, CBS silencing in DSCs (**F**) improves mitochondrial oxygen consumption rate (OCR) and (**G**) restores mitochondrial Complex IV activity. Western blotting for the mitochondrial protein Tom 20 served as a mitochondrial isolation quality control in (**A**). *, ** shows a difference between CC and DSC (* *p* < 0.05; ** *p* < 0.01); ^#, ##^ shows the effect of CBS silencing in DSCs (^#^ *p* < 0.05; ^##^ *p* < 0.01). Reproduced by permission from [90].

In the early 2000s, Kamoun observed an elevation of H_2_S metabolites in the circulation and urine of Down syndrome individuals [269,270] and hypothesized that overproduction of H_2_S by CBS may induce some of the clinical signs of DS [271]. According to the “Kamoun Hypothesis”, supraphysiological H_2_S levels in various cells and tissues induce a form of “metabolic poisoning”, at least in part due to suppression of cytochrome c oxidase (mitochondrial Complex IV) activity and impairment of aerobic ATP generation, which, in turn, produces a global energetic deficit in Down syndrome individuals, culminating in various obvious functional impairments such as reduced exercise tolerance and impaired neuronal functions [271]. The Kamoun Hypothesis is, indeed, consistent with the well-established inhibitory effect of H_2_S on cytochrome c oxidase [87,272,273], the role of H_2_S as a neurotoxic agent and as a mediator that can impair neuronal development [274,275,276,277] as well as with multiple lines of prior studies demonstrating the presence of mitochondrial dysfunction in Down syndrome [278,279,280,281]. While the elevation of H_2_S production in Down syndrome has subsequently been repeatedly confirmed [90,282], the actual functional role of CBS-derived H_2_S in the pathogenesis of mitochondrial dysfunction remained untested until 2019, when our group has directly tested the hypothesis by evaluating the effect of CBS silencing (as well as the effect of AOAA, a PLP-dependent enzyme inhibitor with limited selectivity for CBS, see below) on the proliferation, mitochondrial oxygen consumption and Complex IV activity in Down syndrome fibroblasts. We observed that CBS silencing improves bioenergetic functions, restores Complex IV activity, and these effects culminate in an improved viability and proliferative rate of these cells [90] (Figure 9). 

In an independent line of studies, Herault and his co-workers demonstrated that overexpression of CBS, on its own (i.e., in the absence of the other hundreds of genes that are also dysregulated in Down syndrome) produces neurobehavioral impairments in mice that resemble the phenotype observed in Down syndrome mice [259]. In addition, in a mouse model of Down syndrome (Dp(17Abcg1-Cbs)1Yah), a mouse which carries an extra copy of the mouse chromosome 17 fragment that encodes CBS—as well as several additional genes), neurobehavioral deficits were also observed, and they were ameliorated by CBS silencing [259] (as well as by the action of disulfiram, which is generally viewed as an aldehyde dehydrogenase inhibitor, but which was identified, in the same study, based on phenotypical screens, as a cell-based inhibitor of CBS activity; see below).

The above observations should be considered to be first steps towards directly testing the hypothesis that CBS inhibition may be beneficial in Down syndrome in a clinical setting. The potential role of the CBS/H_2_S pathway in Down syndrome, and potential experimental and clinical approaches focusing on CBS inhibition and/or H_2_S scavenging have recently been reviewed [283,284]. 

### 4.2. Cancer 

Tumors reprogram cells and microenvironment to gain immortality and grow relentlessly. An important cell machinery hijacked for this purpose is the metabolic system, adapted to maintain energy and redox balance. Cancer cells up-regulate various metabolic and energetic pathways to support their increased metabolic rate [285]. The list of these pathways includes the up-regulation of CBS protein [73,82,83,88,89,286,287,288,289,290,291,292,293,294,295,296,297,298,299,300,301,302,303,304,305,306,307,308,309,310,311,312,313,314,315,316,317,318,319]. The cancer types where CBS up-regulation—in many cases, with a documented increase in the intratumoral H_2_S levels) [88,89,233]—has been demonstrated are listed in Table 2. In these studies, multiple tumor tissues from patients and cancer cell lines have been tested for CBS expression, and revealed increasing mRNA and/or protein levels compared to adjacent normal tissue or non-malignant equivalent cells. CBS up-regulation in tumor cells sometimes occurs in combination with up-regulation of other H_2_S-producing enzymes (CSE, 3-MST); in other forms of cancer it is not CBS but one or more of the other H_2_S-producing enzymes that becomes up-regulated [320,321,322,323,324,325]. 

**Figure 10:**
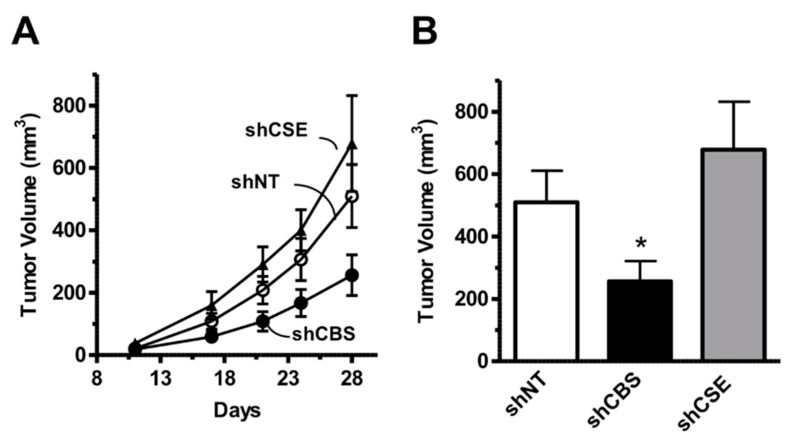
CBS contributes to the maintenance of proliferation of colon cancer cells in vivo. The shRNA-mediated CBS silencing (shCBS) of HCT116 cells attenuates their growth rate after subcutaneous transplantation into nude mice. In contrast, silencing of CSE (shCSE) does not suppress the proliferation of HCT116 cells. Control HCT116 cells were subjected to a non-targeted (NT) shRNA sequence (shNT). * *p* < 0.05 shows significant inhibitory effect of CBS silencing. Reproduced by permission from [88].

Using CBS silencing, several studies have investigated the functional role of CBS in various cancer cells. According to the results of these studies, CBS is involved in the regulation of cell proliferation, mitochondrial bioenergetics and cell viability. In colon cancer cells, CBS silencing resulted in a reduction of basal cellular respiration, ATP synthesis, maximal respiration and spare respiratory capacity in vitro, and the reduction of tumor growth and angiogenesis in vivo [88] (Figure 10). These findings were subsequently confirmed in ovarian cancer cell lines, and it was also noted that CBS silencing produced a marked decrease in cellular glutathione levels as well as an increase in cellular ROS levels [89]. The mechanism by which CBS supports cellular bioenergetics in cancer cells is related, at least in part, to a direct donation of electrons to the mitochondrial electron transfer chain [87,88,310,320,321]. Not only mitochondrial function is regulated in cancer cells in a CBS-dependent fashion, but also mitochondrial morphology. Instead of the fused and elongated mitochondria observed in ovarian cancer cells, CBS-silenced cells presented with fragmented mitochondria lacking network, associated with mitofusin-2 down-regulation. In addition, cristae formation was reduced in CBS-silenced breast cancer cells with vacuolated mitochondria, and there was evidence for mitochondrial permeability transition pore opening [89,306]. Finally, mitochondrial DNA integrity is also regulated by CBS: CBS silencing impaired mitochondrial DNA integrity and reduced the rate of mitochondrial DNA repair [299]. In addition, CBS silencing induced dilation of endoplasmic reticulum and increased cytosolic calcium concentration [294].

CBS silencing also affects the interaction of cancer cells with their microenvironment. For instance, CBS-silenced colon and ovarian cancer xenografts induce less tumor angiogenesis than wild-type cancer cells [88,89], consistently with the known angiogenic role of H_2_S. Moreover, when breast cancer cells are cocultured with activated macrophages, CBS silencing in the breast cancer cells increases the antitumor efficacy of the macrophages [294], consistently with the known cytoprotective role of H_2_S. There are several lines of evidence to indicate that CBS in tumor cells promotes epithelial-to-mesenchymal transition (which, in turn, increases invasiveness and metastatic potential), while CBS silencing can prevent or partially reverse this process [314,319].

The question whether CBS is regulated as cancer cells assume a more aggressive phenotype has been explored by several investigations. In colon carcinoma and liver cancer cell lines, various insults including oxidative stress, radiation, and chemotherapeutic exposure (collectively termed “potentially lethal damage”) produced an up-regulation of CBS, which, in turn, conferred a protective and more invasive phenotype to the tumor cells [291,293,300,307,310,314,317]. 

CBS silencing can exert additive or synergistic effects with anticancer therapies. For instance, in ovarian cancer, CBS silencing enhanced the efficacy of cisplatin to suppress ovarian cancer xenograft growth, nodule formation and vascularization [89]. Moreover, CBS silencing increased the anticancer efficacy of doxorubicin and sunitinib in HepG2 cells, while forced CBS overexpression protected BEL-7404 cells against these anticancer agents [312]. Interestingly, in other instances, suppression of CBS may be actually the underlying mechanism by which certain anticancer approaches exert their effects. For instance, in lung cancer cells, CBS was found to be down-regulated by the up-regulation of ribosomal protein L3 when treated with the common chemotherapy drug 5-fluorouracil; this was associated with the inhibition of cell migration and invasion [298]. MicroRNA 6852 (MIR6852) was also found to regulate the expression of CBS and regulate lung cancer cell ferroptosis [326].

While from the above results it is clear that CBS contributes to the pathobiology of various tumor cells, its up-regulation occurs in combination with a multitude of other biochemical changes in the cancer cell. The logical follow-up question, therefore, is to determine whether the forced up-regulation of CBS in a non-tumorigenic cell can confer a tumor-like phenotype. This question was directly addressed by forced overexpression of CBS into the non-transformed colonic epithelial cell line NCM356 [303]. The presence of CBS in these cells promoted cellular bioenergetics (including switching the cells to prefer anabolic metabolism); enhanced cellular proliferation and invasiveness, resistance to anoikis, and CBS-dependent tumorigenesis in immunocompromised mice. CBS overexpressing NCM356 xenografts produced larger local tumors than wild-type control NCM356 cells, but metastasis was not observed. Metabolomic analysis revealed many differentially expressed metabolites clustered into the glycolytic pathway, nucleotide sugars, pentose phosphate pathway, and lipogenesis. CBS up-regulation also induced broad changes in the NCM356 cell transcriptome with over 350 differentially expressed genes related to glycolysis, hypoxia, and invasive cellular phenotype (e.g., genes regulated by NF-κB, KRAS, p53, and Wnt signaling, genes down-regulated after E-cadherin knockdown, and genes related to increased extracellular matrix, cell adhesion, and epithelial-to-mesenchymal transition) [303]. The same study also revealed that CBS up-regulation is a fairly early process in colonic carcinogenesis: up-regulation of CBS was documented in human biopsies of precancerous adenomatous polyps [303]. 

Taken together, CBS overexpression significantly contributes to the pathogenesis of various cancer cells, and CBS silencing (on its own, or in combination with chemotherapeutic agents or immunotherapy) can exert significant antitumor effects in vitro and in vivo. Based on these findings, coupled together with the anticancer effects of various pharmacological CBS inhibitors (see below) it can be concluded that pharmacological inhibition of CBS has antitumor therapeutic potential. However (see also below), the options are limited with respect to CBS inhibitor compounds that would be potentially suitable for translational work and potential clinical testing.

## 5. Pharmacological Inhibitors of CBS

### 5.1. The “Classical CBS Inhibitor”: Aminooxyacetate 

#### 5.1.1. Discovery and Early Studies 

The importance of the CBS/H_2_S pathway in human pathophysiology has only emerged over the last decade. Before the role of CBS/H_2_S in cancer biology had emerged, and before the re-merging role of CBS/H_2_S in Down syndrome (see Section 2), there was no pharmacological reason to inhibit CBS (only to activate or reactivate it, for the treatment of homocystinuria, see above). Therefore, the field of CBS inhibitors is still in its infancy. Despite multiple recent small-molecule screening campaigns [327,328,329,330] seeking to discover novel, potent, and selective CBS inhibitors, the 100+ years-old “dirty drug” aminooxyacetic acid (AOAA), remains the most commonly used CBS inhibitor to date. AOAA appears to be one of the few CBS inhibitors available to date that is suitable (with a lot of caveats and limitations, see below) for cell-based as well as in vivo biological studies. 

AOAA (also known as O-(carboxymethyl) hydroxylamine or U-7524), is a small molecule belonging to the carboxylic acid family. The compound was originally synthesized by Werner in 1893 [331,332]. Its chemical synthesis was later optimized by Borek and Clarke, whereby acetoxime is condensed with sodium chloroacetate, followed by acid hydrolysis of the resulting acetone carboxymethoxime [333]. AOAA was initially employed as a chemical tool for the isolation of carbonyl compounds, such as ketones or aldehydes. As described by Anchel and Schoenheimer, its aminooxy moiety (ONH_2_) reacts strongly with carbonyl groups thus forming an oxime derivative [334]. The free carboxylic group of AOAA confers the resulting oxime an acidic character which precipitates under acidic conditions, and it can be quantitatively separated by centrifugation. Eventually, the parental ketone (or aldehyde) can be regenerated in the presence of an excess of pyruvic acid which has a high reactivity for AOAA [334]. This method has been successfully used for the separation of carbonyl compounds from biological fluids and tissues [335,336,337]. Indeed, the propensity of AOAA to undergo oximation reactions has been exploited to obtain oxime derivative with therapeutic potential [338,339]. 

Probably the first publication reporting the pharmacological effects of hydroxylamino compounds in a biological system is a report published in 1937, when Mayer and co-workers demonstrated its activity as a bacteriostatic and antibiotic agent [340,341]. In fact, the use of AOAA has been patented after World War II as a supplement to routine aseptic techniques in order to avoid contamination of therapeutic compositions, and its efficacy has been proven on a large range of bacteria, including pathogenic species such as *S. aureus* and diphtheroids [342]. Dienes and colleagues reported that in combination with penicillin, AOAA proved to be effective in converting typhoid bacilli into their L-form (a condition in which these organisms are partially or completely cell wall deficient) and inhibiting their growth [343]. The mechanism of AOAA’s antibacterial effects was unclear in these studies. It was suggested that the mechanism relates to AOAA’s propensity to combine with ketones and aldehydes in living cells; the finding that AOAA’s pharmacological effect could be reversed by the addition of pyruvate was consistent with this hypothesis [344]. The exact mechanism of AOAA’s antibacterial action was never definitely clarified, but it is worth mentioning that recent studies have re-emphasized the biological importance of bacterial H_2_S-producing enzymes in various bacterial functions including antibiotic resistance and resistance to elimination by the immune system, and, indeed, AOAA has been demonstrated to exert antibacterial effects, especially in combination with antibiotics or immune cells. The mode of AOAA’s action, according to these reports, is, at least in part, related to the inhibition of bacterial H_2_S production by the bacterial CBS homologs [345,346,347].

A key advance in understanding the mode of AOAA’s pharmacological action came from studies exploring the reaction of AOAA with vitamin B6 (pyridoxine) derivatives such as pyridoxal. AOAA (as well as related compounds such as hydroxylamine, hydrazine or semicarbazide) proved marked reactivity against the aldehydic moiety of pyridoxal, thus forming the corresponding oxime in aqueous solutions at acidic pH [348,349]. It has been hypothesized that such reactions may also contribute to AOAA’s antibacterial effects. More importantly, however, the AOAA-pyridoxal-interactions have led to the recognition that AOAA can also covalently bind to PLP, which functions as an important coenzyme in a large number of enzymatic processes, catalyzing decarboxylation, deamination, transamination, racemization, β- and γ-eliminations and substitutions, retro-aldol and Claisen reactions and others [350,351]. Thus, AOAA and other carbonyl-trapping agents started to emerge as potentially useful pharmacological tools for inhibiting PLP-dependent enzymes [352]. The first report in which AOAA was identified as an inhibitor of a PLP-dependent enzyme is a paper by Wallach who (working at the Upjohn Company) in 1961, described the inhibitory effect of AOAA on the catalytic activity of γ-aminobutyric acid (GABA) aminotransferase (GABA-T) in vitro and in vivo [353]. Over the subsequent decade, the inhibitory effect of AOAA on several different PLP-dependent enzymes has been demonstrated, including alanine transaminase [354], glutamate decarboxylase [355], alanine racemase [356], histidine decarboxylase [357], D-amino acid transaminase [358], aspartate transaminase [359,360,361] and DOPA- (levodopa or l-3,4-dihydroxyphenylalanine) decarboxylase [362]. The inhibitory effect of AOAA on CBS was first described by Braunstein and colleagues, in the context of discovering that CBS is identical with another enzyme that was previously termed and characterized as an AOAA-inhibitable enzyme termed “serine sulfhydrase” [363]. Several years later, it was also noted that another enzyme in the transsulfuration pathway, CSE is also inhibited by AOAA [364]. 

#### 5.1.2. The Mode of AOAA’s Inhibitory Effect: the AOAA-PLP Interaction 

PLP, a catalytically active cofactor of various enzymes including both enzymes of transsulfuration pathway CBS and CSE, exists in tautomeric equilibrium between the inactive enolimine and the active ketoenamine forms. PLP owes its great versatility to its ability to form Schiff bases with α-amino moiety of amino acids, thus stabilizing reaction intermediates [362,365,366]. Commonly, the access to the active site of PLP-dependent enzymes is very narrow. As described in 3.1 (see above), in CBS, PLP is buried in a structural cavity between the N- and C-terminal domains, where it is locked into the active site by linking the ε-NH_2_ group of Lys119 via Schiff base, thus forming an internal aldimine. Moreover, the PLP ring is further anchored in the active-site pocket thanks to a dense net of hydrogen bonds, for instance between the 3’ hydroxyl group of PLP and Nγ2 of Asn149 or between the phosphate group and Thr257 and Thr260 [18,366]. A key step of catalytic mechanism of PLP is the displacement of Lys119 and the formation of a new Schiff base between PLP and the aminoacidic substrate [367]. 

**Figure 11:**
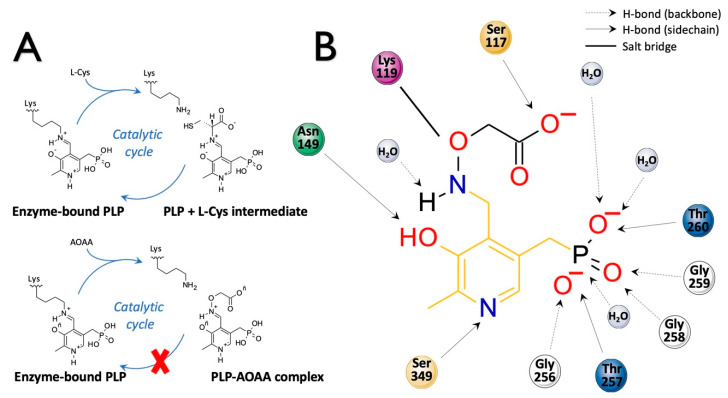
The proposed mechanism of AOAA’s action on human CBS. (**A**) Two representative intermediates of the CBS reaction mechanism. The cofactor is bound to the ε-amino group of Lys119, thus forming an internal aldimine. The interaction with the substrate (e.g., L-cysteine) forms an external aldimine followed by internal rearrangement and eventually the regeneration of the enzyme-bound PLP. The interaction of AOAA stop the catalytic cycle through irreversibly binding the formyl moiety of PLP, thus preventing the regeneration of enzyme-bound PLP. (**B**) Docking simulation: interactions of the tentative PLP-AOAA complex in the catalytic pocket of CBS (Part B is reproduced by permission from [11]).

Several lines of evidence suggest that the mechanism of action of AOAA and other carbonyl-trapping reagents involves the formation of an irreversible Schiff base with the PLP cofactor thus preventing the regeneration of an enzyme-bound PLP, as shown, for instance, on CSE using absorption and fluorescence spectroscopy [364]. The same reaction pattern has been described in several other transaminase in which AOAA has been used as a suicide inhibitor [362]. This mechanism of action is further supported by crystal structures of PLP-dependent enzymes complexed with AOAA [368]. However, Braunstein and colleagues observed that CBS was not inhibited by DL-cycloserine, a strong inhibitor of many transaminases (including CSE), thus suggesting that the CBS inhibition mechanism might be different [363]. The basis of this difference might be the fact that CSE and CBS belong to two different fold type families of PLP-dependent enzymes [369]. So far, the study of the mechanism of action of putative inhibitors has been challenging, since the heme moiety of the human CBS interfere with the shift absorption spectrum of PLP intermediates. Recently, the inhibitory mechanism of a hydrazine derivative has been characterized working on yeast CBS, a form naturally lacking heme and thus suitable for spectroscopic study. The authors suggest a model according to which the PLP-inhibitor complex form a hydrazone, which then undergo some internal rearrangements and eventually leaving the cofactor in the pyridoxamine form, which is catalytically inactive [370]. Our proposed mechanism of interaction between AOAA and PLP in the active site of CBS is shown in Figure 11.

The localization of PLP is different in different PLP-dependent enzymes, depending on the structure of the enzyme and its active site. Therefore, the access of AOAA (or other carbonyl-trapping reagents) is not uniform to all PLP groups in all PLP-dependent enzymes. Accordingly, AOAA does not inhibit the activity of all PLP-dependent enzymes, and even when an inhibitory effect occurs, the potency of the inhibition can be markedly different (see also below). Usually, the PLP active site is hidden in a narrow cleft of the protein architecture, therefore low molecular weight compounds (such as AOAA) tend to be more suitable for this purpose than larger molecules. Conversely, it should be also stressed that only some (but certainly not many or not the most) of the known PLP-dependent enzyme inhibitors inhibit CBS. In a screening campaign seeking to identify novel CBS inhibitors [329], we have assembled a collection of PLP-dependent inhibitors, which included the ornithine decarboxylase inhibitor DL-difluoromethylornithine; the thymidylate synthase, dihydrofolate reductase and glycinamide ribonucleotide formyltransferase inhibitor pemetrexed, the GABA transaminase inhibitor vigabatrin, the GABA transaminase and aromatic L-amino acid decarboxylase inhibitor 3-hydroxybenzylhydrazine and the DOPA decarboxylase inhibitor carbidopa. The majority of these compounds did not exhibit significant CBS-inhibitory effects, with the exception of 3-hydroxybenzyl-hydrazine, which inhibited CBS activity with an IC_50_ of approximately 60 µM and carbidopa, which was a marginal inhibitor of CBS activity (6% inhibition at 100 µM) [329]. 

#### 5.1.3. Effects of AOAA in Mammalian Cells and Tissues In Vitro and In Vivo

As discussed above, initial work related to the pharmacological effects of AOAA in cells, tissues, and animals presumed that the mode of action is its inhibitory effect on GABA-T. This work was, in fact, initiated at the Upjohn Company. Based on the extensive preclinical work and the early-stage clinical work (see below) with the compound, the original intention of the Upjohn Company—which designated AOAA as “U-7524”—must have been to develop and market the compound as a CNS therapeutic: specifically, as an anticonvulsant. In vertebrates, GABA is mainly known as an inhibitory neurotransmitter of CNS and low GABA levels have been associated with epileptic seizures and convulsions. Early in vivo studies in different animal models indicated that treatment with AOAA decreases seizure susceptibility, an effect which was assumed to relate to increased brain concentration of GABA. When administered orally, subcutaneously, or intravenously, AOAA was found to cross the blood–brain barrier to yield CNS concentrations sufficient to inhibit GABA-T. Thus, based on various preclinical studies [353,371,372] AOAA has been proposed as a potential drug suitable for treatment of neurological diseases associated with decreased GABA levels.

Human safety studies with AOAA (i.e., “U-7524”) were conducted by Upjohn in the early 1960s using daily doses of up to 400 mg [372,373,374]. AOAA was well tolerated with nausea, vomiting, dizziness, and fatigue noted, without any clinical side effects and minor laboratory findings (slight increases in serum transaminase levels). The first human clinical efficacy study with AOAA was conducted at the Central Islip State Hospital, New York, USA, with initial findings published in the early 1960s [374]. Eight patients with associated seizure disorder were given U-7524 (orally, in doses extending from 150 to 300 mg/day) in addition to their previously prescribed anticonvulsant medication. The initial findings were striking. There was a significant decrease in seizure frequency in all patients with complete abolishment of convulsions in 50% of the study subjects. In addition, in patients who continued to have seizures, the duration of their episodes was abbreviated. The effects of AOAA were also associated with neurobehavioral improvements. A similar small-scale pilot efficacy study (8 epileptic patients with mental retardation, aged 5-37 years, treated with 200 mg/day AOAA given in 4 divided doses), suggested the potential anticonvulsant efficacy of AOAA [375]. A follow-up published in the Journal of Canadian Medical Association, describing the results of a study conducted in a pediatric population suffering from “syndrome of infantile massive spasms with mental deterioration”, where once again, approximately 50% of the AOAA-treated children were found to exhibit a suppression of seizure incidence and severity [373]. In an article published in the journal “Therapeutic Trends” in 1963, AOAA was listed as one of the new drug development candidates of the Upjohn Company [376]. However, after these initial studies, we were unable to find any further industry-sponsored clinical trials with AOAA; the pharmaceutical development of this compound must have been discontinued. 

Nevertheless, investigator-initiated clinical studies with AOAA continued throughout the 1970s and early 1980s with publications appearing until the early 1990s. These studies were no longer associated with the Upjohn Company and used chemical grade materials (e.g., chemical product produced by the Eastman Chemical Company and placed into capsules by the physician investigators involved in the study). In one such study carried out in the late 1970s, it was tested whether AOAA can alleviate symptoms associated with the excessive GABA production in patients affected by Huntington disease (HD) [377,378]. This study used the “No Observed Adverse Effect Level” of orally administered AOAA to 2.5 mg/kg/day as established in normal volunteers, thus representing approximately 50% of the doses used in the preceding epilepsy studies in the 1960s. However, treatment of HD patients with AOAA did not produce any detectable clinical benefit in this study. Some of the observed side effects were similar to the prior observations (dizziness, drowsiness, vomiting) but also ataxia and psychotic behavior were also noted. Moreover, a marked increase in plasma levels of proline and hydroxyproline were noted, suggesting an interference of AOAA with other enzymes than GABA-T [378]. In another clinical trial in adult patients, AOAA (200–400 mg/day, given in 4 divided doses) has been shown to induce a reversible loss of hearing sensitivity and has been proposed to be potentially useful in the palliative treatment of tinnitus [379,380]. (This development direction was based on preclinical studies implicating GABA-T in the regulation of cochlear function and studies demonstrating that AOAA can induce a temporary hearing loss in guinea pigs). However, the conclusion of a follow-up tinnitus study was that the efficacy of AOAA was too low and the incidence of side effects was too high for further considering the clinical development of AOAA for tinnitus [379,380]. With the strengthening of the regulations around clinical trials, investigator-initiated trials of this type were no longer possible in the 1990s. This change, as well as the emerging questions around the efficacy, safety (see above) and specificity (see below) of AOAA, led to a complete stop to clinical testing of AOAA. This is, actually, not surprising, given the compound’s lack of selectivity. Moreover, based on biochemical measurements, the oral bioavailability of AOAA appears to be low, although, to our knowledge, actual plasma or tissue AOAA levels have never been reported in the published literature. Based on the changes in CNS GABA levels in response to i.v., s.q. or oral administration of AOAA [353], one can estimate that AOAA’s oral bioavailability is likely between 10 and 20% in rats (while in humans, to our knowledge, its oral bioavailability has never been determined). 

As mentioned earlier, some of the earliest pharmacological effects of AOAA were demonstrated in bacteria, where the compound exerted antimicrobial effects. Over subsequent years, the effects of AOAA and other aminooxy compounds have extended to several other microorganisms and demonstrated antibacterial and antimycobacterial actions of the compound. For instance, in *M. tuberculosis* AOAA displayed a moderate efficacy in cell growth inhibition (IC_50_ in the order of tens of micromolar). The proposed mechanism of action relies on the interference with the methionine regeneration pathway via inhibition of a branched-chain amino acid aminotransferase (BCAT), an enzyme involved in the catabolism of branched-chain amino acids such as leucine, isoleucine and valine [381,382,383]. Moreover, AOAA was shown to inhibit the proliferation in *P. falciparum*, thus suggesting a further application as antiprotozoal [383,384,385,386,387]. These effects were attributed to AOAA’s effects on the aminoacidic cycle via inhibition of aspartate aminotransferase (also known as glutamic oxaloacetic transaminase or GOT), although a recent study carried out on *T. gondii* reproduced the same effect on KO-GOT models, suggesting the additional involvement of a (currently unidentified) GOT-independent enzymatic pathway [387].

AOAA has also been widely used as malate-aspartate shuttle (MAS) inhibitor in various in vitro and in vivo studies [388,389,390,391,392,393,394] by targeting GOT activity. GOT has recently emerged as a pivotal enzyme in the maintenance of cancer metabolism through both stimulating cell bioenergetics, at least in part through glutamine metabolism [395,396,397], glutamine the latter being a metabolic fuel of high rate proliferating cells [395,398]. Accordingly, AOAA (in this context, solely or primarily viewed as a GOT inhibitor) has been shown to suppress the bioenergetic function and the proliferation of various cancer cells (Table 3). 

#### 5.1.4. AOAA as a “CBS Inhibitor” (or a Broad Inhibitor of H_2_S Biosynthesis) In Vitro and In Vivo

**Figure 12:**
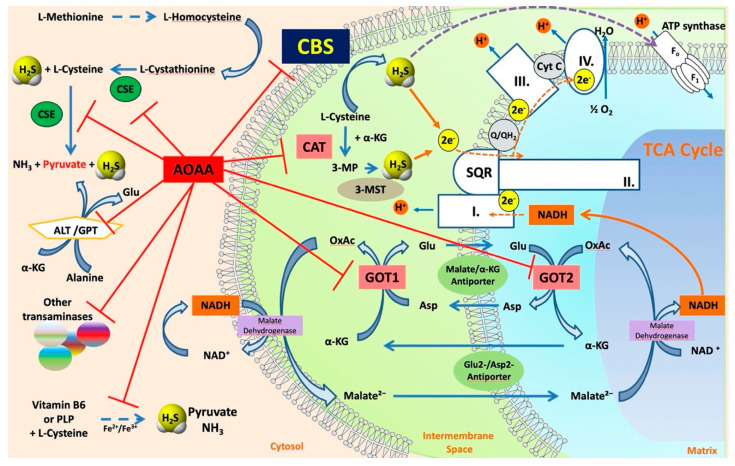
Multiple modes of AOAA’s action on H_2_S producing pathways and other transaminases in cancer cells. AOAA suppresses cellular H_2_S levels by directly inhibiting CBS and CSE activity, by suppressing H_2_S formation through the 3-MST pathway via inhibition of cysteine amino transferase (CAT), and by inhibiting the non-enzymatic formation of H_2_S from vitamin B6 or PLP. In addition, AOAA also inhibits a variety of transaminases (including GOT1, a key enzyme of the malate/aspartate shuttle). In a cancer cell, these combined effects of AOAA may produce synergistic inhibition of cellular bioenergetics, resulting in an impairment of cancer cell proliferation and viability. By inhibiting CBS-derived and 3-MST-derived H_2_S, AOAA suppresses mitochondrial electron transport and cancer cell bioenergetics by preventing the donation electrons at complex II, by suppressing the H_2_S-induced direct stimulation of ATP synthase and by lifting the H_2_S-mediated inhibition of intramitochondrial adenylate cyclase (this latter effect is not shown on this scheme). The malate-aspartate shuttle translocates electrons that are produced in glycolysis across the semipermeable inner membrane of the mitochondrion to support oxidative phosphorylation. These electrons enter the electron transport chain at Complex I. The shuttle system is required because the mitochondrial inner membrane is impermeable to NADH (a primary reducing equivalent of the electron transport chain). In humans, the cytoplasmic enzyme (GOT1) is one of the key enzymes in the malate shuttle: it catalyzes the interconversion of aspartate and α-ketoglutarate to oxaloacetate and glutamate using PLP as a cofactor. By inhibiting GOT, AOAA reduces the transfer of electron donors to the mitochondria, thereby providing an additional mode for the suppression of cancer cell bioenergetics. Finally, many tumors up-regulate their metabolism through glutaminolysis. In this process, glutamine is taken up into the cells, and it rapidly deaminated by deaminases to yield glutamate (the uptake and the conversion is not shown in the current scheme). In turn, glutamate (Glu) is converted by alanine aminotransferase (ALT) enzymes, in particular by glutamate pyruvate transaminase 2 (GPT2) to α-ketoglutarate and enters the TCA cycle. Because AOAA inhibits ALT/GPT2, this process is inhibited, and the tumor cells become deprived from an important metabolic fuel. The current figure is a modified version of a figure that was reproduced by permission from [11].

As discussed above, CBS is only one of the many enzymes that are inhibited by AOAA. However, for CBS, AOAA is one of the most potent inhibitors of this enzyme known to date, with IC_50_ of approximately 1–8 µM, depending on the assay conditions used [399]. Importantly, however, AOAA is also a potent inhibitor of a second H_2_S-producing enzyme, CSE (another PLP-dependent enzyme), with an even higher potency (CSE’s IC_50_ is 1 µM in the same report where CBS’ IC_50_ is 8 µM) [399]. Moreover, AOAA can also indirectly inhibit H_2_S formation by the 3-MST system as it can inhibit the enzymatic generation of the 3-MST substrate, 3-mercaptopyruvate (see below). Moreover, AOAA can even inhibit some non-enzymatic pathways of H_2_S formation (see also below). The inhibitory effects of AOAA on various H_2_S biosynthetic pathways (as well as on other transaminases, especially as they affect mitochondrial function and cellular bioenergetics) are depicted in Figure 12. 

Although the selectivity of AOAA clearly does not justify this designation (see above and see also below for further discussion on this subject), over the last decade, in the emerging field of H_2_S biology, AOAA has been often used in various biological experiments and it is commonly referred to as a “CBS inhibitor”. In addition, to be fair, AOAA is, indeed, a CBS inhibitor, and a potent one, but one that has many additional pharmacological effects unrelated to CBS. Accordingly, in vitro and in vivo data confirm that CBS activity is inhibited in cells, tissues and animals after AOAA treatment, and the expected biochemical changes are, indeed, elicited (such as inhibition of H_2_S biosynthesis). The first report, demonstrating that AOAA can inhibit H_2_S biosynthesis in a biological system, dates to 1982. It actually comes from the plant literature and demonstrates that AOAA inhibits H_2_S generation in a variety of plant leaves (*C. sativus, C. pepo, N. tabacum, C. blumei, B. vulgaris, P. vulgaris, M. sativa, H. vulgare, and G. hirsutum*). However, the enzyme involved in H_2_S synthesis has not been defined in this study; it was assumed that it is a “PLP-dependent enzyme” [400].

In mammals, the first evidence demonstrating the inhibitory effect of AOAA, as a CBS inhibitor, on H_2_S biosynthesis was provided by Abe and Kimura in their seminal report published in 1996, where they proposed that H_2_S can act as a mammalian biological mediator and neurotransmitter. In this report, in brain homogenates, the investigators have measured H_2_S production and found that there was a significant basal production, which could be inhibited with AOAA (with an IC_50_ of approximately 10 µM), and it could be enhanced by the allosteric CBS activator SAM [57]. Subsequent studies have demonstrated the inhibitory effect of AOAA on CBS-catalyzed H_2_S synthesis in marine invertebrates [401], as well as in a multitude of mammalian cells and tissues [90,139,143,290,402,403,404,405]. In cell-based systems, the effective concentration of AOAA to suppress H_2_S biosynthesis appears to be cell-type dependent (which may reflect a combination of differential cell uptake and cellular metabolism of the compound); in human fibroblasts, AOAA, already at 3 µM, inhibits H_2_S generation [90] while in most transformed cells (e.g., colon cancer cells or lung cancer cells), AOAA concentrations of 100 µM or above are required to suppress H_2_S production [88,296,299,308]. 

Although the cell and tissue penetration of AOAA is low (see above), and its therapeutic ratio is small, over the last decade, more than 100 in vitro and in vivo studies have been published that used AOAA as a tool to study H_2_S-related pathways. In the majority of these studies, AOAA was referred to as a “CBS inhibitor”; in most of these studies, however, the effect of AOAA on H_2_S production was not directly measured (nor were any other enzymatic pathways assessed that would be also expected to be affected by AOAA). The published pharmacological effects of AOAA that are likely to relate to inhibition of CBS and the associated reduction of cellular H_2_S biosynthesis include inhibitory effects on tumor cell bioenergetics, proliferation and angiogenesis [88,296,299,308] and normalization of CBS-mediated H_2_S overproduction and restoration of normal mitochondrial function in Down syndrome cells [90]. CBS-derived H_2_S may also have a stimulatory effect on platelet aggregation, and AOAA has been shown to counteract it, resulting in an anti-platelet-aggregatory effect—although an inhibitory effect on aspartate aminotransferase may also play a role in AOAA’s action [406,407].

#### 5.1.5. The Lack of AOAA’s Selectivity as a Pharmacological Inhibitor

In recent years AOAA has been usually addressed as a CBS ‘classical’ inhibitor, although many concerns about its selectivity has been arisen as it inhibits many other enzymes, most of them through reactions with their PLP in their active center [362]. We were able to identify approximately 40 enzymes that have been shown to be inhibited by AOAA [7,329,353,357,358,362,363,371,383,400,429,430,431,432,433,434,435,436,437,438,439,440,441,442,443,444,445,446,447,448,449,450,451,452,453,454,455,456,457,458,459,460,461,462,463,464,465,466,467] (Table 4). Please note that the potency of AOAA on these various enzymes is markedly different. This difference likely represents, in some instances, the differences in the experimental conditions employed to determine the potency of the inhibitor, but it is also likely to reflect true inhibitory potency differences. Although these inhibitory effects occur through AOAA-PLP interactions, depending on the enzyme in question, the access of the inhibitor to the PLP prosthetic group is likely different, depending on the structure of each enzyme’s particular active site.

Therefore, the biological activity of AOAA is hard to interpret; it very much depends on the biological context. For instance, in the oncologic field, AOAA’s anticancer effects have been attributed to inhibition of CBS, because of the marked up-regulation of this enzyme in different cancer cells as compared to the surrounding healthy tissues (see above). In any case, it should be taken into consideration that AOAA inhibits other enzymatic H_2_S-synthesizing routes, as well as enzymatic routes that are beyond the H_2_S-associated pathways. With respect to H_2_S biosynthesis, AOAA, in fact, directly or indirectly inhibits H_2_S biosynthesis by all three principal enzymes, CBS and CSE (see above), and, indirectly, 3-MST as well. The latter effect is due to the fact that AOAA is an inhibitor of cysteine amino transferase (CAT; 2.6.1.3), an enzyme that is also known as aspartate aminotransferase or glutamic oxaloacetic transaminase (AST or GOT; 2.6.1.1). This enzyme, on one hand, catalyzes the biosynthesis of L-glutamate from L-aspartate. However, with cysteine as its substrate, the very same enzyme is involved in the biogenesis of 3-mercaptopyruvate, which, in turn, is substrate of 3-MST, an H_2_S- and polysulfide-producing enzyme. Indeed, it has been demonstrated that targeting CAT with AOAA results in impairment of 3-MST/CAT H_2_S mediated route [405]. AOAA may even inhibit non-enzymatic H_2_S biosynthesis: Yang and colleagues reported that H_2_S can also be produced in biological systems by a reaction catalyzed non-enzymatically by free PLP and iron, with cysteine serving as a substrate. This reaction eventually produces pyruvate, NH_3_, and H_2_S. Interestingly, the reaction is potently inhibited by AOAA, which reacts with the aldehyde group of PLP, thus preventing its interaction with cysteine [468]. Thus, we must conclude that in the “H_2_S Universe”, AOAA should be designated as an “inhibitor of H_2_S production”, rather than a “CBS inhibitor”.

However, this designation, still, does not consider the dozens of mammalian enzymes that are also inhibited by AOAA (Table 4), many of which exhibit a broad cell and tissue expression patterns in mammals. For example, if we return to the discussion concerning cysteine aminotransferase/aspartate aminotransferase (see above) it should be emphasized that when, aspartate is used as a substrate, this enzyme catalyzes the production of oxaloacetate, thus bridging the Krebs cycle with the urea cycle and gluconeogenesis through the malate/aspartate shuttle (MAS). In fact, in a separate field of biochemistry (that almost never communicates with the “H_2_S Universe”), AOAA has been often employed to pharmacologically inhibit GOT to modulate the above metabolic pathways; in this context, AOAA has been found to suppress cellular metabolism in various experimental contexts ranging from cardiovascular disease to cancer [88,296,320,369,389,394,423,426,469,470,471] (see also: Table 3). Figure 12 depicts some key AOAA-inhibitable metabolic pathways (in the “H_2_S Universe” and beyond) and their potential synergistic interactions in support of tumor cell bioenergetics.

Several other metabolic changes are also modulated by AOAA. For instance, as shown in liver cells isolated from Wistar rats, 0.2 mM AOAA significantly affected ethanol metabolism in a way which cannot be explained with transaminase inhibition [361]. In fact, Meijer and Van Dam issued a warning about the use of AOAA when working with ethanol metabolism, as AOAA (but not D,L-cycloserine, another PLP-dependent enzyme inhibitor) chemically reacts with acetaldehyde, an oxidation product of ethanol [466]. Likewise, when one of the substrates of the reaction is pyruvate, it is highly recommended adding the inhibitor before pyruvate, ensuring an efficacious inhibition of the transaminase [466,467]. Indeed, its marked propensity to react with carbonyl group can be a double-edged sword, as ketones and aldehydes can reverse inhibition of PLP-dependent enzymes by AOAA [467]. 

In summary, the various biological effects of AOAA, over the years, have been attributed to a wide range of enzymatic targets and associated biochemical pathways (Table 3 and Table 4). In some cases, some biochemical changes associated with the presumed molecular target of AOAA have been explored directly (i.e., in ex vivo studies) or indirectly (e.g., in parallel, complementary in vitro pharmacological and molecular biological studies). 

Although it is unlikely that the effects of AOAA in any cell or any in vivo experimental model can be attributed to any single enzymatic target (or a pathway regulated by this target), it is likely that the anticonvulsive effects of AOAA are mainly due to inhibition of GABA-T and the consequent accumulation of GABA in the CNS. With respect to the inhibitory effect of AOAA on cancer cell metabolism, the two best supported theories relate to inhibition of tumor cell GOT1 and glutaminolysis and inhibition of the mitochondrial effects of CBS-derived H_2_S. Since both pathways, ultimately, culminate on cellular bioenergetic processes, it is possible that these two pathways work in an additive or synergistic fashion (Figure 12). 

However, other targets of AOAA should also not be discounted. For instance, AOAA can also suppress kynurenine synthesis [445,446] and kynurenine has been implicated in the regulation of tumor cell metabolism and tumor immunity [472,473,474].

### 5.2. Potentially Repurposable CBS Inhibitors 

#### 5.2.1. Benserazide 

Since the beginning of the 20^th^ century, hydrazine (*R*-*NH*-*NH*_2_) and aminooxy (*R*-*ONH*_2_) derivatives have been used mainly as carbonyl-trapping reagents for analytic purposes. However, this was not the case of carbohydrazides (*R*-*C*(=*O*)-*NH*-*NH*_2_), which, despite the marked reactivity towards aldehydes and ketones, had the disadvantage to split spontaneously in aqueous solutions, thus preventing their use in the separation process [334,475]. Following the emerging role of carbonyl-trapping agents in biological systems [340,341], an increasing interest surrounded hydrazides for their marked propensity to induce convulsions [476,477]. Interestingly, the widely used anti-tuberculosis drug isoniazid and its parental hydrazine proved to induce a range of side effects when administered at high doses, including convulsions, peripheral neuropathy, and pellagra, most of them associated with vitamin B_6_ antagonism [478]. Because of the known chemical reactivity of hydrazides and hydrazines towards the formyl PLP moiety, the relationship between hydrazide/hydrazine-induced convulsions and PLP-depending enzymes inhibition was soon established, unveiling the great potential of these compounds in modulating nervous system signaling [479,480,481]. As expected with carbonyl-trapping compounds, their effect was reduced in the presence of relevant endogenous ketones, such as pyruvate, α-ketoglutarate or oxaloacetate, therefore raising some concerns about their selectivity [482]. 

The above findings about the putative role of hydrazide derivatives to inhibit the activity of PLP-requiring enzymes, inspired chemical screening campaigns to identify novel DOPA decarboxylase inhibitors. At that time, the knowledge of the metabolism of catecholamine was still in its infancy, but the pivotal role of DOPA decarboxylase in their biosynthesis was already well-recognized, as it has been first described by Peter Holtz in 1939 (as reported by Blaschko in “A half-century of research on catecholamine biosynthesis” [483]). The inhibitors known at the time had significant disadvantages, because the exhibited potent inhibitory effects not only towards DOPA decarboxylase but also interference with monoamine oxidase [371,484,485,486,487,488]. 

Benserazide, known also as seryl-trihydroxybenzylhydrazine or Ro 4-4602 (originally synthesized by Dr B. Hegedüs, F. Hoffmann-LaRoche, Basel, Switzerland) proved to be a potent inhibitor of DOPA decarbocylase, without affecting monoamine oxidase in the brain [489,490]. In a study in which male Wistar rats were subjected to intraperitoneal injection of labeled L-DOPA alone (control animals) or 50 mg/kg benserazide followed by labeled L-DOPA (treated animals), it was reported that the concentration of cerebral catecholamines in treated animals was almost 60 times higher than in controls. Conversely, in peripheral tissues catecholamines concentration of treated animals was less than half as compared to controls, indicating a selective inhibition of DOPA decarboxylase in extracerebral organs. From a qualitative point of view, cerebral titrated catecholamines consisted mainly of dopamine (80–90%), thus suggesting a pharmacological employment of benserazide in neurological disorders such as Parkinson’s disease [491]. This finding was further corroborated by Tissot and colleagues [492]. Subsequent clinical trials in Parkinsonian patients confirmed the effectiveness of the co-administration of L-DOPA + benserazide (ratio 4:1). This drug combination was commercialized in 1975 as Madopar^®^ by Hoffmann-LaRoche and allowed to decrease the equivalent dose of L-DOPA to 1/5, thus reducing the L-DOPA associated side effects (typically gastrointestinal intolerance and in some cases cardiac arrhythmias) [492,493,494,495].

The revelation that benserazide also acts as a CBS inhibitor is relatively recent. The CBS-inhibitory action of the compound emerged in two independent screening campaigns aimed at the identification of compounds that inhibit CBS-derived H_2_S biosynthesis [327,329]. In the first screening campaign, the inhibitory potency was relatively low: its IC_25_ (i.e., not IC_50_!) value was determined as 125 µM [327]. However, in a subsequent screening campaign, benserazide exhibited a significantly higher inhibitory activity towards human recombinant CBS (IC_50_ = 30 µM) [329]. (Many compounds tend to degrade upon storage in chemical libraries, and we have noticed that benserazide is particularly unstable compound prone of oxidation at room temperature. We suspect that the reason for the lower potency in the initial screen was due to such degradation). 

Using in silico docking simulation, a model was proposed whereby benserazide binds to the PLP coenzyme, thus forming a reversible but stable Schiff base with the aldehydic group of PLP [329]. Although AOAA has a potency 10-fold higher than benserazide on recombinant CBS enzyme in vitro, benserazide was much more effective at impairing cellular bioenergetics and proliferation rate (IC_50_ 20 µM) than AOAA (IC_50_ 300 µM), as seen on HCT116 colorectal cancer cell line (most likely due to its better cell uptake) [329]. In a subsequent in vivo study, benserazide (50 mg/kg/day) delayed the growth of HCT116 tumor xenografts in a nude mouse model [329]. Interestingly, as opposed to the effect of AOAA, which lost a significant part of its antiproliferative efficacy in a multi-drug resistant clone of HCT116 human colon cancer cells, benserazide remained partially effective in multi-drug-resistant cancer cells [309].

Since benserazide is a clinically used compound, the idea of repurposing benserazide as a potential agent tool for the experimental therapy of diseases with CBS overexpression might be a theoretical possibility. Since benserazide is not readily CNS-permeable, Down syndrome is a less attractive indication than perhaps some forms of cancer. One of the many questions, of course, is whether benserazide in patients could be given in sufficiently high concentrations to inhibit CBS activity. The CBS-inhibitory IC_50_ values shown above are not particularly encouraging, but the cell-based activity of the compound is, nevertheless, present in low micromolar concentrations. The fact that Parkinson’s patients treated with benserazide showed increased homocysteine plasma levels [496,497] is also encouraging as this could be interpreted as a result of CBS inactivation. In rat pharmacokinetic studies benserazide has been shown to achieve plasma 4–40 µM plasma concentrations [498,499]. Further studies would be desirable to assess whether benserazide may be repurposed as a potential candidate for the modulation of CBS activity in vivo. In this context, nevertheless, a few facts related to benserazide should be kept in mind: (a) benserazide is not available or approved anywhere in the world as a stand-alone compound; it is only available as a component of the two-component drug combination, Madopar (see above); (b) Madopar is only used in certain countries in Europe and in Canada and the UK; in other countries, other combinations of L-DOPA and DOPA decarboxylase are used. For instance, in the USA, various L-DOPA/ carbidopa combinations are approved. (However, carbidopa—in contrast to benserazide—is not a significant CBS inhibitor).

#### 5.2.2. 2,3,4-Trihydroxybenzylhydrazine, an Active Metabolite of Benserazide 

In the original study which tested the in vitro and in vivo biological action of benserazide as a DOPA decarboxylase inhibitor, the compound 2,3,4-trihydroxybenzylhydrazine (also known as Ro 1-5127) was also evaluated (and compared to its seryl derivative, benserazide). In vitro, both compounds proved to be potent inhibitors of DOPA decarboxylase with IC_50_ values of 0.02 µM and 0.04 µM, respectively. However, while trihydroxybenzylhydrazine was immediately reactive towards DOPA decarboxylase, its seryl derivative produced a full inhibition of the enzyme only after a 90-minute incubation. This delay in the progression of the enzymatic inhibition suggested that benserazide undergoes a conversion into its hydrazine precursor, likely through hydrolysis of the seryl-hydrazine linkage [490]. 

Spectrophotometric data on the interaction between trihydroxybenzylhydrazine and DOPA decarboxylase are indicative of a binding with the coenzyme at the active site. The type of inhibition seems to be pseudo-irreversible, thus suggesting a further interaction with some other sites of the enzyme beside the PLP binding pocket [500,501]. As shown from the crystal structure of DOPA decarboxylase complexed with carbidopa (a compound structurally similar to benserazide—which, nevertheless, is not a significant inhibitor of CBS), the inhibitory effect of carbidopa, and presumably of benserazide, is due to the formation of an hydrazone linkage with the PLP cofactor through its hydrazine moiety [501]. Although trihydroxybenzylhydrazine represents the actual DOPA decarboxylase inhibitor, its seryl derivative offers some significant pharmacokinetic improvements. Indeed, while in vivo studies showed that both compounds were highly active towards DOPA decarboxylase, only the seryl derivative did not significantly interfere with other enzymes, such as monoamine oxidase, diamine oxidase, catecholamine-O-methyl transferase, transaminase of aromatic amino acids and tryptophan hydroxylase, indicative that the seryl-residue might be responsible for a higher specificity [500].

The same study where we have identified benserazide as a CBS inhibitor [329] we have explored the possibility that its active metabolite, 2,3,4-trihydroxybenzylhydrazine acts as CBS inhibitor and thereby raising the possibility that it is, in fact, this metabolite that is, partially or fully responsible for the pharmacological action of benserazide observed on CBS activity and various cellular functions. The CBS-inhibitory potency of 2,3,4-trihydroxybenzylhydrazine was similar to benserazide (IC_50_ of approximately 30 µM on recombinant human CBS) and—similar to benserazide and other CBS inhibitors—inhibited the proliferation of colon cancer cells in vitro without inducing detectable cytotoxicity, except at relatively high concentrations [329]. Structural modeling studies have indicated that 2,3,4-trihydroxybenzylhydrazine likely interacts with the PLP group in the active center of CBS [329]. 

Interestingly, a structurally related compound, 3-hydroxybenzylhydrazine (also known as NSD-1015) has also been identified as a CBS inhibitor in a screening campaign [329]. This compound is traditionally viewed as a PLP-enzyme inhibitor (that reacts with PLP to form 3-hydroxybenzylhydrazone). It is known as an inhibitor of GABA aminotransferase and L-aromatic amino acid decarboxylase; it is also known to have a good ability for crossing the blood–brain barrier [502]. However (as opposed to benserazide or 2,3,4-trihydroxybenzylhydrazine) it is not a clinically used compound, nor is it a metabolite thereof.

#### 5.2.3. Disulfiram

Disulfiram (known also as tetraethylthiuram disulfide) was first synthetized from thiocarbamide in 1881 [503]. It was successfully employed in the industry of rubber vulcanization to manufacture products such as neoprene [504]. The first clue about its potential biological role was reported by the plant physician E. E. Williams, who, observing workers in a rubber processing plant described disturbs such as weakness, headache and nausea showing up immediately after alcohol intake [505]. Williams recognized that the adverse effects of tetraethylthiuram disulfide and related compounds were somehow “beneficial” in terms of the therapy of alcoholism; the first study on its potential pharmacological application was carried out only 10 years later. Volunteers who had ingested an apparently innocuous dose of disulfiram developed the same symptoms described by Williams after drinking even small amounts of alcohol. This symptomatology was associated with high levels of plasma acetaldehyde found in treated volunteers but not in controls [506]. Later, Jacobsen et al., trying to wash a disulfiram batch which was contaminated with copper, accidentally discovered a new form of disulfiram with better pharmacokinetics, which was the patented with the name of Antabus^®^ [507]. 

The mechanism of action of disulfiram is attributed to the inhibition of aldehyde dehydrogenase (ALDH), which is responsible for the conversion of acetaldehyde, an ethanol metabolite, into acetic acid. Therefore, disulfiram induces an accumulation of acetaldehyde because of ethanol intake. After the absorption, disulfiram is readily converted into its corresponding thiol diethyldithiocarbamate, which is then methylated and oxidized in the liver into S-methyl-*N*,*N*-diethylthiocarbamate-sulfoxide and -sulfone. These metabolites are the actual active compounds that inhibit ALDH through an irreversible carbamylation of the catalytic Cys302 residue [508]. 

In recent years, given the high morbidity and mortality associated with oncological diseases, drug repurposing has received renewed interest. Disulfiram has been identified as a potential anticancer drug in different tumor types, namely prostate cancer, breast cancer, colon cancer, ovarian cancer and pancreatic cancer [509,510,511,512,513,514,515]. In an epidemiological study, the drug’s antitumoral action was evaluated using the data of Denmark’s cancer registry. Patients under treatment with disulfiram (because of alcohol dependency) showed a 34% lower mortality compared to patients who stopped taking disulfiram before the cancer diagnosis. Therefore, disulfiram has been suggested to actively influence the progress of the disease, and this was seen particularly for colon, breast and prostate cancer [516]. In fact, several clinical trials have been completed or are currently ongoing in cancer patients with various therapeutic protocols that incorporate disulfiram as part of various combination therapies (e.g. trials NCT02963051, NCT00742911, NCT03323346 and NCT04265274 in the clinicaltrials.gov database). It is interesting to note that most of the cancer types for which a putative antineoplastic action of disulfiram has been reported are also forms of tumor where CBS has been shown to be overexpressed (see Table 1).

Recently, in a yeast-based screening model (identification of pharmacological compounds that can suppress the development of methionine auxotrophy induced by Cys4 overexpression), disulfiram has been identified as a putative inhibitor of cellular CBS activity [259]. Disulfiram appears to inhibit CBS activity only in a cellular environment (because, presumably, it requires some form of metabolism or bioconversion): from prior screens evaluating the direct effect of various clinically used pharmacological compounds on recombinant CBS in vitro, disulfiram has not emerged as a significant direct inhibitor of CBS activity [329]. 

Although its molecular mode of action is likely complex and likely to involve many additions beyond CBS, it is interesting to note that in the Dp(17Abcg1-Cbs)1Yah model of Down syndrome mice (as discussed above; a model which is associated with increased CBS expression and associated cognitive dysfunction), treatment with disulfiram produced significant neurological benefits [259]. This finding may pave the way for repurposing disulfiram as an inhibitor of CBS activity in vivo, with potential applications in oncological and neurological diseases.

### 5.3. Additional Classes of CBS Inhibitors

#### 5.3.1. Hydroxylamine 

Hydroxylamine, a simple small organic compound with the chemical formula of NH_2_OH, was first synthetized in 1865 by exposing tin and hydrochloric acid to nitric acid ethyl ester [517]. Since the end of the 19^th^ century, similar to many other aminooxy compounds, it has been largely employed for the separation of aldehydes and ketones from biological systems. Its marked nucleophilic character makes it prone to react with carbonyl groups thus producing water insoluble oximes [518,519]. For the same reason, it has been traditionally considered extremely poisonous and just a few attempts to introduce it into medicine were made until the first half of the 20^th^ century. Indeed, because of its putative toxicity it has been described as bacteriostatic agent and inhibitor of photosynthetic reactions in plants [520,521]. The latter has been suggested to take place by hydroxylamine-mediated inhibition of catalase [EC 1.11.1.6] [522]. 

The intuition that carbonyl-trapping reagents may interact with the formyl moiety of pyridoxal, paved the way for its employment as PLP-dependent enzyme inhibitor. In this regard, Baxter and Roberts have widely used this compound as inhibitor of 4-aminobutyrate aminotransferase [EC 2.6.1.19], thus laying the groundwork for the comprehension of the biological role of GABA [523]. However, soon it became clear that the main disadvantage of this drug was the lack of selectivity. Indeed, hydroxylamine has been shown to inhibit several enzymes, many of them transferases, namely alanine transaminase [EC 2.6.1.2] [524], glycine transaminase [EC 2.6.1.4] [525], kynurenine-oxoglutarate transaminase [EC 2.6.1.7] [526], histidinol-phosphate transaminase [EC 2.6.1.9] [527] and ornithine-oxo-acid transaminase [EC 2.6.1.13] [528], just to mention a few. Moreover, hydroxylamine decomposes in aqueous solutions and gives rise to a nitric oxide (NO) [529,530,531], an endogenous vasodilator and cytoprotectant, which lends this compound a whole another dimension of pharmacological action.

Based on its pharmacological character outlined above, it is not surprising that hydroxylamine is also an inhibitor of CBS (as well as CSE); it reacts with the PLP prosthetic group to form an oxime [363,366]. In fact, it shows some selectivity for CSE over CBS [399]. Therefore, it is quite surprising that some of the published literature—including several recent reports—refers to hydroxylamine as a “CBS inhibitor” [57,157,160,532,533,534].

Nevertheless, it has been known for over 50 years that hydroxylamine exerts anticancer effects in a variety of experimental models in vivo [535,536,537]; the current interpretation of these findings must be that this effect is most likely the combined action of hydroxylamine on various transaminases and other enzymatic targets including its inhibitory effect on H_2_S biosynthesis. The pharmacological action of hydroxylamine on a broad range of enzymes makes its biological action difficult to interpret, therefore it can be considered a possible tool for proof-of-concept in vitro enzymatic assays rather than an in vivo pharmacological tool.

#### 5.3.2. Copper 

Copper is the second most abundant transition metal in biological systems after iron. It is involved in different processes of cellular physiology, but it can also be noxious, depending on the concentration [538]. Its ability to act as an enzymatic cofactor for several reactions has been thoroughly studied. The metal center of copper-dependent proteins can be classified into different types, according to the mode of copper-protein binding. For instance, the Cu-S(Cys) bond is characteristic of the type I copper center, while in the case of the CuA center a methionine is involved [539]. When the concentration of this micronutrient exceeds a threshold, the cellular homeostasis may be threatened. 

One of the proposed mechanisms through which copper can interfere with the activity of various enzymes is through the interaction with methyl-thiol or thiol moiety of aminoacidic residues harbored in the active site or in other sites relevant for the enzymatic activity [540,541]. This raises the opportunity to use this micronutrient to inactivate a target enzyme, although, as reasonably can be deducted from the postulated mechanism, the selectivity of this approach might be a major concern. 

The finding that copper inhibits H_2_S biosynthesis is at least half a century old. The first study was carried out on CSE, in which case, among the heavy metals tested for their inhibitory effect, Cu (II) proved to be one of the most effective (but cadmium and mercury were also inhibitory), whereas zinc was ineffective [542]. Cu (II) has been reported to inhibit CBS as well [543] and, on a molar basis, it is the most potent inhibitor of CBS known to date; in a recent study its IC_50_ was established as 0.3 µM [329]. However—and this has also been known for over a century—copper can also directly interact with H_2_S [544], and in assays where CBS activity is assessed by the measurement of H_2_S production, the potency of copper may be overestimated because of this reaction.

Although the exact mechanism of copper’s CBS-inhibitory effect has not been fully elucidated, one can speculate that the oxidation state of the CBS redox sensing motif 272CXXC275 may play a role [315]. 

Even though copper is a potent inhibitor on isolated or recombinant CBS enzyme in vitro, a significant reduction of HCT116 cell line proliferation rate was achieved only at concentration of 1 mM, presumably because of its low cellular uptake [329]. Copper itself cannot be considered a pharmacological tool to target CBS, because of its obvious toxicity. However, it would be interesting to follow up on approaches that may selectively induce intracellular mobilization and/or testing copper-containing molecules that may deliver copper intracellularly (and, potentially, in a targeted manner for CBS), thereby reducing the drug dose and consequently the unspecific binding. In fact, various classes of copper-containing compounds have been tested in recent years as potential anticancer therapeutics [544,545,546,547,548,549], although, in most cases, the exact molecular mode of their action is complex and has only been partially characterized. 

#### 5.3.3. NSC67078

One of the first screens published to identify CBS inhibitors was a 2013 report by Zhou and colleagues, who used a high-throughput tandem-microwell assay and screened over 20,000 compounds in order to identify novel inhibitors of CBS-derived H_2_S synthesis [328]. Several polycyclic ketone-based selective inhibitors emerged from the screen, perhaps the most interesting being NSC67078 (1,6-dimethyl-pyrimido[5,4-e]-1,2,4-triazine-5,7(1H,6H)-dione), a compound that was previously also referred to in the literature as toxoflavin, xanthothricin, or PKF118-310. The compound was reported as a competitive inhibitor of CBS with an IC_50_ of 12 µM, with some selectivity for CBS over CSE. A structurally related compound, NSC11041 exhibited a slightly higher potency on CBS, but it showed no selectivity for CBS over CSE. Both compounds were subjected to computer modeling and they were found to fit well into the CBS active site. Also, the inhibitory effect of these compounds could be concentration-dependently inhibited by excess PLP in the CBS assay [328].

In a subsequent screen, we have used NSC67078 as a positive control CBS inhibitor. The potency of the compound on CBS was found to be approximately 30 µM [329]. However, to our surprise, it not only potently inhibited the CBS-induced H_2_S response (quantified by the fluorescent H_2_S sensor AzMC), but also the H_2_S donor GYY4137-induced AzMC fluorescence. This suggested that part of the inhibition of the CBS-induced signal is due a scavenging or quenching effect, and not a direct enzymatic inhibition. HCT116 cell proliferation was potently and concentration-dependently inhibited by the compound, consistent with its action on CBS (in this cell line, CBS silencing also exerts a marked inhibition of cell proliferation; see above). 

The specificity of this compound as a CBS inhibitor is limited, because the compound is also known to act as a potent inhibitor of the β-catenin pathway [550,551,552,553]. Moreover, it also inhibits SIRT1/2 [554] as well as KDM4A (lysine demethylase 4A) [555]. In fact, the history of this compound goes back to the 1930s when the compound (originally termed toxoflavin), was identified as the cause of food poisonings in a region of Indonesia [556]. Toxoflavin has been shown to be a toxin of bacterial origin (*P. cocovenenans*) being involved in wasting of a range of cereals and vegetables, and currently is considered a threat to global rice production [557]. Structurally, the molecule belongs to the family of pyrimidotriazine and it is considered a potent broad-spectrum antibiotic [558]. However, it is also a potent toxin in mammals; for example, in mice, it displays an LD_50_ of 2–8 mg/kg [559]. It is likely that its toxicity relates to disturbing the mitochondrial electron chain and to the intracellular generation of ROS [560,561]. 

Thus, similarly to most of the compounds discussed in the above sections, the anticancer effects of NSC67078 are most likely the result of a combination of pharmacological effects: perhaps CBS inhibition contributes to it, but most likely so does β-catenin inhibition, SIRT inhibition, histone post-translational modifications and subsequent changes in chromatin organization, changes in cellular redox balance and perhaps additional pharmacological actions as well. 

#### 5.3.4. Sikokianin C 

Niu and colleagues conducted a high-throughput screening, employing a fluorescent thiol to capture the CBS-catalyzed production of methanethiol (CH_3_SH) from the artificial substrate methylcysteine. CBS inhibitors identified (from a library of 6000+ natural compounds) included the polyphenol sikokianin C and several related compounds as CBS inhibitors, some of which exhibited some selectivity for CBS over CSE [562]. Based on the results of previous screens, we can conclude that not only sikokianin C but also many other flavones and polyphenols (including tannic acid, tangeretin, alpha-mangostin and others) are CBS inhibitors [327,329]. 

In a subsequent publication, Niu and colleagues followed up on the pharmacological effects of sikokianin C in biochemical models (molecular docking with CBS) and in cell-based and animal models of colon cancer [302]. The molecular docking indicated that there are five residues in CBS (His203, Tyr308, Tyr223, Asn194 and Thr193), which may interact with the phenolic hydroxyl groups and the carbonyl group of sikokianin C to form five hydrogen bonds. Niu and colleagues have also tested the inhibitory effect of sikokianin C in various CBS mutants; the inhibitory effect of the molecule diminished, and the molecular modeling predicted less interactions with the enzyme. 

Follow-up studies evaluated the inhibitory effect of sikokianin C on the proliferation of HT29 cells (a human colon cancer cell line expressing high levels of CBS). The inhibitor decreased the proliferation of HT29 cells, and its antiproliferative effect was attenuated (but was not abolisghed) in HT29 cells with siRNA-mediated CBS silencing. As expected, the silencing of CBS, on its own, slowed down the baseline proliferation rate of the tumor cells. 

The fact that sikokianin C exerts residual effects in the absence of CBS, and the finding (also reported in the same paper) showing that sikokianin C also inhibits the proliferation of NCM356 cells (a relatively normal colon epithelial cell line with low CBS expression; see also above) indicates that the antiproliferative effect of sikokianin C must be the net effect of multiple pharmacological effects (only one of which is CBS inhibition). Indeed, sikokianin C has been reported in the literature to exert a variety of pharmacological effects including antimalarial effects, anti-inflammatory effects and an inhibitory effect on the expression of inducible nitric oxide synthase (iNOS) [563,564,565]. 

Although its mode of action is likely mixed, in a model of tumor-bearing mice, sikokianin C exerted dose-dependent inhibitory effects, and appeared approximately equally efficacious with AOAA (50% inhibition of HT29 tumor growth in a mouse xenograft model) [302]. 

#### 5.3.5. CH004 

A high-throughput screen conducted by Zhou and colleagues using a tandem-microwell assay [328] (see above) has identified the hit molecule 3-benzyl-1,6-dimethylpyrimido[5,4-e][1,2,4]triazine-5,7(1*H*,6*H*)-dione (designated as “CH004”), which was not disclosed in the original publication, but was the focus of a follow-up report published in 2018 [256]. This compound (other than, perhaps, copper; see above) may be the most potent inhibitor of CBS known to date, with an IC_50_ less than 1 µM. CH004 also shows an approximately 30× selectivity for CBS over CSE. It should be noted that CH004 (similar to copper and probably many of the polyphenols) also has a direct H_2_S scavenging activity; the signal in response to authentic (chemically generated) H_2_S is inhibited by the compound with an IC_50_ of approximately 70 µM [313]. These findings initially may suggest that CH004 is substantially more potent as a CBS inhibitor than as a H_2_S scavenger. However, it must be noted that in the H_2_S scavenging assay a fast-release H_2_S donor was used, and not a slow-release compound, and the choice of the H_2_S donor will have a significant effect on the IC_50_ value of the scavenger; one can predict that its scavenging potency would be higher if a slow-acting H_2_S donor, for instance GYY4137 would have been used, as in other counterscreens, e.g., [329].

The anticancer effect of CH004 was tested in several cell lines that express high levels of CBS protein (HepG2, HEK293T, Huh7, H22, Panc-28, HCT116, and MDA-MB-231). In all cases, the molecule inhibited cell proliferation with IC_50_ values in the 10-20 µM range; in the same concentration range, cellular H_2_S generation was also suppressed. In HEK293T cells with siRNA-mediated CBS silencing, the proliferation-inhibitory efficacy of CH004 decreased, but was not completely abolished. As expected, the silencing of CBS, on its own, slowed down the baseline proliferation rate of the tumor cells. As discussed with respect to sikokianin C in the previous section, the fact that CH004 exerts residual effects in the absence of CBS suggests that the antiproliferative effect of CH004 must be the net effect of multiple pharmacological effects (only one of which is CBS inhibition). 

The finding that CH004 increases ROS generation, a finding that was interpreted by the investigators as an indicator of ferroptosis, may be consistent with the regulatory role of CBS in tumor cell ferroptosis; independent studies using CBS silencing [317] are consistent with this conclusion. However, the finding that CH004 arrests the cell cycle at the S phase are not consistent with the cellular roles of CBS in tumor cells, since no evidence of S-phase arrest was observed in tumor cells in response to CBS silencing and cancer cell S-phase arrest was also not noted with other small molecules (e.g., AOAA) [296]. Rather, this action is more likely to be related to CBS-independent pharmacological effects of CH004.

Interestingly, CH004 has lost its inhibitory effect in the cells expressing the Q222A CBS mutant, but hydroxylamine retained its inhibitory effect on this mutant enzyme [313].

In contrast with the high potency of CH004 on recombinant CBS and in cultured cells, in the in vivo studies using tumor-bearing mice, the efficacy of the molecule was not particularly impressive: a dose of 10 mg/kg/day partially inhibited the growth of liver tumor xenografts [313].

#### 5.3.6. 6S and Related Inhibitors 

A common strategy adopted in rational drug design is the synthesis of compounds structurally resembling the physiological interactors (e.g., substrates) of the target enzyme. In the case of CBS, these are L-serine, L-cysteine, L-homocysteine, and (L,L)-cystathionine. Considering that the latter displays a K_m_ one order of magnitude lower than the other substrates, cystathionine may represent an interesting structural template to explore for medicinal chemistry modifications. Recently, McDune and colleagues synthetized and tested cystathionine analogs both in vitro and in vivo for their ability to inhibit CBS [566]. From the structural point of view, the authors functionalized cystathionine derivatives with -NHNH_2_, -ONH_2_ or -NHOH moieties in place of the α-amino group to obtain tighter binding to the formyl group of PLP. Moreover, they replaced the central thioether with an isosteric olefinic bond (C = C). The final products were a series of symmetric molecules displaying two carbonyl-trapping moieties, in which a C = C ‘zips’ the two halves of the drug. In vitro studies performed on the truncated form of CBS (lacking the SAM-binding domain) revealed that a derivative designated as “6S” was the most potent among all the molecules evaluated (Ki ∼48 µM) and exhibited some selectivity towards CBS over CSE. 6S inhibited H_2_S production in cell lysates (Ki ∼50 µM) and protected against the H_2_S-associated cell damage in human neuroblastoma SH-SY5Y cell line overexpressing CBS. 

The efficacy of 6S was also confirmed in vivo. In a rat stroke model, intracerebroventricular injection of 6S (1.6 μmol/kg) reduced the size of the infarct and attenuated microglial activation in the reperfusion phase [566]. Overall, although 6S is not a particularly potent CBS inhibitor, it shows some CBS selectivity and in vitro and in vivo activity. Therefore, it can be considered a potentially useful lead compound for the development of more effective inhibitors.

#### 5.3.7. Additional CBS Inhibitors

Table 5 summarizes all the CBS inhibitors discussed in the previous sections, and includes several additional molecules that have emerged from the various CBS screens. Although aurintricarboxylic acid appears to be fairly potent as a CBS inhibitor, its specificity and potential practical utility is likely low, because this compound has the propensity for polymerization in aqueous solution, forming a stable free radical that has been shown to inhibit various protein-nucleic acid interactions. Accordingly, this compound has previously been shown to possess several pharmacological activities, including protease inhibition, complement inhibition, ribonuclease inhibition and neuraminidase inhibition [567,568,569]. Nevertheless, a recent screening campaign identified this compound as a potent inhibitor of another H_2_S-producing enzyme, CSE [570]. 

The other compounds listed in the final part of Table 5 have emerged from the various screening campaigns already discussed. However, many of these compounds have not been further characterized for selectivity and/or for cellular and in vivo actions.

## 6. Conclusions and Future Directions

In the above sections we have attempted to compile, organize and interpret the massive amount of information which has accumulated over many decades on the role of CBS in health and disease, especially with respect to conditions associated with CBS induction/activation and direct and indirect means to inhibit the activity of this enzyme. Although CBS is traditionally viewed as an enzyme primarily responsible for the metabolism of homocysteine, more recent work realized another important function of this enzyme as one of the principal sources of H_2_S, a gaseous biological mediator with multiple regulatory roles in the vascular, nervous, and immune system. There have been several decades of work related to inactivating CBS mutations as the cause of classical homocystinurias; novel approaches related to the experimental therapy of homocystinuria (e.g., enzyme replacement therapy) have now entered clinical trials. However, this stand-alone field of CBS mutations and homocystinuria was only briefly discussed in the current article. Instead, we have primarily focused on the mechanisms and consequences of CBS up-regulation, in particular in the context of Down syndrome and cancer. In these conditions, the preclinical data indicate that inhibition or inactivation of CBS exerts beneficial effects; progress in this field is expected to stimulate further work to identify clinically useful and sufficiently selective inhibitors of CBS. It should be also mentioned that future potent and selective competitive CBS inhibitors could be used to improve folding and to stabilize active conformations of many pathogenic CBS mutant, where protein misfolding was identified or implied as a cause of loss of CBS activity and homocystinuria, and, perhaps paradoxically, they may in fact act as CBS activators or reactivators under such circumstances (as discussed in [188]). 

With respect to pharmacological inhibitors, the current article provides the historical background and the state-of-the-art pharmacological action of the existing small molecules that are termed “CBS inhibitors” (starting with the “classical compound” AOAA and continuing with the various classes of more recent CBS inhibitors, typically identified from medium-throughput academic screening campaigns). Many of the compounds that emerged from these campaigns are not ideal with respect to their medicinal chemistry properties and their specificity/selectivity profile. 

Further studies, screening larger libraries must be conducted to determine whether CBS is, in fact, an “undruggable” enzyme, or whether small molecules with good pharmacological properties can be discovered in the future. The information provided in the current review highlights the fact that the currently known compounds, although termed “CBS inhibitors”, can only be used with extreme caution to study the biological roles of CBS. Given the wide range of their pharmacological effects on many other experimental systems, the currently available CBS inhibitors must be applied with extreme caution in experimental settings and the results should be interpreted with the above considerations in mind. Appropriate pharmacological controls (e.g., testing the reversibility of the CBS inhibitors’ biological effects with H_2_S donors) or molecular controls (e.g., using CBS-deficient cell and animal models), as discussed previously [11] should always be employed. Moreover, CBS inhibitors of various structural classes (with diverse secondary effect profiles) should be employed in well-controlled pharmacological experiments.

In conclusion, we must admit that, even after a decade of work, we are not much closer to identify CBS inhibitors that would be suitable for translational work or clinical trials. At the same time, the science has progressed to the point where Down syndrome and several forms of cancer can be considered to be “validated targets” for intensified translational efforts for the discovery and development of pharmacological CBS inhibitors. Our comprehensive overview of direct and indirect approaches to inhibit the enzyme should inspire further advances in this area.

## Figures and Tables

**Table 1:** Potential indirect approaches for inhibition of CBS activity. Please note that most of these approaches are only theoretical and all of them are expected to induce broad-based cellular side effects well beyond the regulation of CBS.

Principal Approach	Effect on CBS	Pharmacological Modulators	Potential Off-Target Effects
Inhibition of SAM binding to CBS	Partial inhibition of CBS catalytic activity. Destabilization of the CBS tetramer	MAT inhibitors, competitors of SAM binding to CBS (SAM analogs)	Inhibition of other SAM-dependent enzymes and processes
Stimulation of CO or NO binding to CBS	Partial inhibition of CBS catalytic activity	NO or CO donors	Activation other NO- or CO-dependent enzymes and processes (e.g., vasodilatation, hypotension)
Inhibition of CBS phosphorylation	Partial inhibition of CBS catalytic activity	Kinase inhibitors	Inhibition of other enzymes phosphorylated by the same kinase; modulation of multiple downstream processes
Inhibition of CBS S-glutathionylation	Partial inhibition of CBS catalytic activity	Glutathione S-transferase inhibitors	Inhibition of other enzymes glutathionylated by the same S-transferase
Stimulation of SUMOylation	Proteolytic degradation of CBS; reduced total cellular CBS activity	Possible approach may be the modulation of upstream processes, e.g., E1 activating enzyme (the heterodimer SAE1/2) or E2 conjugase (Ubc9). No known pharmacological agents	Broad dysregulation of protein processing and protein degradation
Stimulation of ubiquitination	Proteasomal degradation of CBS; reduced total cellular CBS activity	Pharmacological activation of E1 activating, E2 conjugating and E3 ligating enzymes (only theoretical; no known inhibitors). Or: pharmacological inhibition of deubiquitinases (this approach has been proposed to degrade undruggable targets for cancer therapy)	Broad dysregulation of protein processing and protein degradation
Inhibition of CBS proteolytic cleavage	Inhibition of the proteolytic conversion of CBS into the highly active 45-kDa form; inhibition of cellular CBS activity	Proteolysis inhibitors (not suitable as a practical approach; selective intracellular delivery of protease inhibitors is not feasible)	Broad dysregulation of protein processing and protein degradation
Activation of Lon protease	Proteolytic degradation of CBS into inactive forms	Lon activators (e.g., Heat Shock Protein Q) exist but only as experimental tools	Broad dysregulation of mitochondrial protein homoeostasis
Inhibiting substrate availability by blocking cystine transport into the cell	Lower CBS activity	Cystine/glutamate antiporter system blockers	Broad dysregulation of sulfur-containing amino acid homeostasis

**Table 2:** Up-regulation of CBS and functional effect of CBS silencing in various cancers. ^a^ Problematic cell lines, contaminated. Originally thought to originate from a normal fetal liver, shown to be a HeLa derivative [295]. Abbreviations: A549, human lung adenocarcinoma cell line; A2780, human ovarian endometrioid adenocarcinoma cell line; BEAS 2B, normal human bronchial epithelial cell line; BPH-1, prostatic epithelial cells derived from benign human prostatic hyperplasia; Calu-6, human lung adenocarcinoma cell line; CPT, camptothecin; CP20, human papillomavirus-related endocervical adenocarcinoma cell line; DLD-1, human colon adenocarcinoma cell line; DOX, Doxorubicin; DU145, androgen-dependent human prostate cancer cell line; EGI-1, human cholangiocarcinoma cell line; ER, endoplasmic reticulum; FTE188, fallopian tube-derived epithelial cells; GB-D1, human gallbladder carcinoma; GB-H3, human gallbladder carcinoma cell line; GOx, glucose oxidase; HCT116, human colon carcinoma cell line; HepG2, human hepatoblastoma cell line; HMEC, human mammary epithelial cells; HMCL, human myeloma cell line; HOSE, human ovarian surface epithelial cells; HT29, human colon adenocarcinoma cell line; HUCCT-1, human intrahepatic cholangiocarcinoma cell line; HUH-28, human intrahepatic cholangiocarcinoma cell line; H522, human lung adenocarcinoma cell line; H1944, human lung adenocarcinoma cell line; Kuramochi, human high grade ovarian serous adenocarcinoma cell line; LNCaP, androgen-dependent human prostate cancer cells; LoVo, human colon adenocarcinoma cell line; MCF-7, estrogen receptor positive human breast cancer cells; MCF-10 A, normal human breast cells; MDA-MB-468, estrogen receptor negative human breast cancer cells; MFN2, mitofusin-2; MPTP, mitochondrial permeability transition pore; mtDNA, mitochondrial DNA; OSE, normal human ovarian surface epithelial cells; NCM356, normal human colonic epithelial cell line; OVSAHO, human high grade ovarian serous adenocarcinoma cell line; OV90, human ovarian adenocarcinoma cell line; OV167, human ovarian serous adenocarcinoma cell line; OV202, human ovarian serous adenocarcinoma cell line; PPCL, human polyclonal plasmablastic cell line; RCC4, clear cell renal cell carcinoma cell line; ROS, reactive oxygen species; rpL3, ribosomal protein L3; RWPE-1, normal human prostatic peripheral epithelial cells; SHSY5Y, neuroblastoma cell line; SNU 245, human cholangiocarcinoma cell line; SNU 308, human gallbladder carcinoma cell line; SNU 1079, human intrahepatic cholangiocarcinoma cell line; SKOV3, human ovarian serous cystadenocarcinoma cell line; TFK-1, human cholangiocarcinoma cell line; TykNu, human high grade ovarian serous adenocarcinoma cell line; U-87 MG, human glioblastoma-astrocytoma cell line; WPMY-1, normal human prostatic myofibroblast stromal cells; 5-FU, 5-fluorouracil.

Cancer Type	Evidence for CBS Up-regulation	Effect of CBS Silencing	Reference
Biliary tract carcinoma	CBS cRNA hybridization levels measured on oligonucleotide microarray are higher in gallbladder carcinoma, intrahepatic cholangiocarcinoma, distal bile duct carcinomas, EGI-1, TFK-1, HUH28, HUCCT-1, SNU 245, SNU 308, SNU 1079, GB-H3, and GB-D1 cells than in normal extrahepatic biliary and gallbladder epithelial scrapings	Not tested	[287]
Breast cancer	• CBS mRNA levels detected by RT-qPCR and protein levels detected by immunoblotting are both higher in Hs 578T, MCF-7, and MDA-MB-468 cells than in HMEC and MCF-10A cells • CBS protein levels detected by immunohistochemistry are increased with the disease progression on tissue microarray with 60 human breast cancer tissue, and compared to the controls human breast epithelial tissue • CBS protein levels detected by immunoblotting are higher in doxorubicin-resistant MCF-7 cells that in normal MCF-7 cells	• Silencing of CBS in MCF-7 and MDA-MB-468 cells reduces cell viability in the presence of GOx, DOX or activated macrophages • Silencing of CBS in MCF-7 cells reduces xenograft growth in female Balb/c nude mice • Silencing of CBS in MCF-7 and MDA-MB-468 cells causes dilation of the ER and increases cytosolic calcium concentrations • Silencing of CBS in MCF-7 and MDA-MB-468 cells decreases cristae formation and increases vacuole formation in mitochondria, increases MPTP opening, and decreases mitochondrial reserve capacity	[82,289,294]
Colon cancer	• CBS protein levels detected by immunoblotting are higher in human colorectal tumor tissues compared to respective normal mucosa tissues • CBS protein levels detected by immunoblotting are higher in LoVo, HCT116, and HT29 cells compared to NCM356 cells • CBS protein levels detected by immunoblotting are higher in premalignant polyps compared to normal mucosa tissues • CBS protein levels detected by immunohistochemistry are increased in hyperplastic polyps compared to normal crypt cells • CBS protein levels detected by immunohistochemistry on tissue microarray are increased in 40 human colon cancer tissues compared to paired adjacent tissues over 52 colorectal cancer cases • CBS protein levels are increased in the colon cancer cell derived circulating tumor cell population CTC-MCC-41 • The development of multi-drug resistance is associated with an up-regulation of CBS protein in HCT116 cells	Silencing of CBS in HCT116 cells decreases cell proliferation and cellular *bioenergetics* in vitro and attenuates HCT116 xenograft growth and vascularization in female Balb/c nude mice	[88,290,296,300,302,303,308,309,310,313,315,318]
Glioma	CBS mRNA levels detected by PCR and protein levels detected by immunoblotting are both higher in U-87 MG cells than in SHSY5Y cells	• Not tested	[301]
Liver cancer	• CBS protein levels detected by immunoblotting are higher in HepG2 cells and SMMC-7721 than in HL-7702 cells ^a^ • In HepG2 cells stress conditions (e.g., oxidative stress, chemotherapeutics, irradiation) induces cancer cell stemness and multi-drug resistance and this is associated with up-regulation of CBS protein	• Silencing of CBS in SMMC-7721 decreases cell viability and proliferation, increases ROS levels and apoptosis	[291,293,297,312,314]
Lung cancer	• CBS mRNA levels detected by RT-qPCR and CBS protein levels detected by immunoblotting are higher in human primary tumor tissues compared to matched human normal tissues • CBS protein levels detected by immunoblotting are higher in human lung adenocarcinoma tumors than in normal adjacent tissues • CBS protein levels detected by immunoblotting are higher in A549, H522, and H1944 cells compared to BEAS 2B cells	• Down-regulation of CBS by rpL3 enhances Calu-6 cells apoptosis and reduces cell migration and invasion • Transient CBS depletion represses mtDNA repair and increases CPT-induced necrosis in A549 cells	[298,299,319]
Multiple myeloma	• cRNA hybridization levels for CBS, measured on oligonucleotide microarray, are higher in human malignant plasma cells from patients with multiple myeloma than in normal plasma cells • mRNA levels detected by RT-qPCR are higher in HMCL than in PPCL	Not tested	[286]
Ovarian cancer	• Human ovarian tumor tissues exhibited moderate-strong CBS protein expression detected by immunohistochemistry on tissue microarray • CBS mRNA levels detected by RT-qPCR and protein levels detected by immunoblotting are both higher in OV167, OV202, SKOV3, and A2780 cells than in OSE cells • CBS protein levels detected by immunoblotting are higher in OV90, CP20, OVSAHO, Kuramochi, and TykNu and cisplatin-resistant TykNu cells than in OSE, FTE188 and HOSE cells • The ferroptosis inducer small-molecule erastin induced an up-regulation of CBS protein and yielded an erastin-resistant version of ovarian cancer cell lines SKOV3 and OVCA429.	• Silencing of CBS in OV202, SKOV3, A2780, and cisplatin-resistant A2780 decreases total cellular glutathione level and cell proliferation • Silencing of CBS in A2780 cells increases cellular and mitochondrial ROS levels, down-regulates NF-κB, decreases cellular bioenergetics, and sensitizes to cisplatin • Silencing of CBS in cisplatin-resistant A2780 reduces xenograft growth and vascularization, and nodules formation in female nude mice, enhances sensitivity to cisplatin, and decreases MFN2 expression • Silencing of CBS in CP20 and OV90 decreases cell proliferation, mitochondrial membrane potential, and network by promoting mitochondrial fission, cellular bioenergetics and promotes MFN2 degradation • In the erastin-resistant version of ovarian cancer cell lines SKOV3 and OVCA429, CBS silencing induces cell death via induction of ferroptosis	[89,292,306,307,317]
Prostate cancer	• CBS protein levels detected by immunoblotting are higher in BPH-1, LNCaP, and DU145 cells than in RWPE-1 and WPMY-1 cells • CBS protein levels measured by immunofluorescence are higher in LNCaP cells than in RWPE-1 cells	Not tested	[83]
Renal carcinoma	• CBS protein levels detected by immunohistochemistry are increased with the disease progression on tissue microarray, and in 53 renal urothelial carcinomas and 9 renal clear cell carcinomas at Fuhrman grade IV compared to 11 benign renal cortex tissues • cRNA hybridization levels of CBS, measured on oligonucleotide microarray are higher in 2 angiomyolipoma and 3 papillary carcinoma tissues compared to respective unaffected part of kidney tissues	Not tested	[304,305]
Bladder cancer	CBS protein levels were detected in bladder tissue specimens (gallbladder squamous cell/adenosquamous carcinomas and adenocarcinomas) and in the bladder carcinoma cell lines 5637, EJ, and UM-UC-3	Not tested	[311]
Thyroid cancer	Increased CBS protein levels were detected in thyroid carcinomas compared to benign thyroid tissue (but not in thyroid follicular adenomas or oncocytomas)	Not tested	[73]

**Table 3:** Pharmacological effects of AOAA in various in vivo experimental models and the proposed underlying pharmacological mechanism(s) action. The listed studiess [88,175,178,296,299,320,394,396,408,409,410,411,412,413,414,415,416,417,418,419,420,421,422,423,424,425,426,427,428,429] provides a selection of the in vivo physiology and efficacy studies obtained with AOAA in various animal studies over the last six decades.

Animal Model	Dose of AOAA	Effects of AOAA; Proposed Mechanism of Action	Reference
Methionine sulfoximine or thiosemicarbazide induced seizures in mice, Sprague-Dawley rats, and cats	23–50 mg/kg i.p. single dose	AOAA dose-dependently decreased the incidence of convulsions and improved survival. The mechanism of action was proposed to be inhibition by AOAA of GABA-T activity in the CNS and subsequent elevation of brain GABA content; in support of this hypothesis, brain GABA levels were measured and were found to be increased at the same doses of AOAA where functional benefits were also noted.	[408]
Endocochlear potentials in response to 6 kHz tone bursts in anesthetized guinea pigs	10–80 mg/kg i.v. single dose	AOAA dose-dependently attenuated the generation of endocochlear potentials. The mechanism of action was not identified, but observations of this type have subsequently led to clinical trials with AOAA in patients with tinnitus.	[409]
Isonicotinic acid hydrazide-induced seizures in male Swiss albino mice	23 mg/kg i.p. single dose	AOAA dose-dependently decreased the incidence of convulsions. The mechanism of action was proposed to be due to a combined inhibition by AOAA of GABA-T activity (which inhibits GABA degradation) and of glutamate decarboxylase activity (which catalyzes GABA production from glutamate), and the resulting changes in the brain GABA content are the function of these two combined enzymatic effects.	[410,411]
Pentobarbital metabolism in mice	30 mg/kg i.v. single dose	AOAA increased pentobarbital plasma levels and decreased the plasma levels of pentobarbital metabolites. The mechanism of action was not identified, but it was suggested to relate to an AOAA-induced broad suppression of cellular bioenergetics.	[412]
Cobalt-induced epilepsy in male piebald rats	2.5–10 mg/kg i.p. single dose	AOAA reduced the frequency of epileptic spikes in the secondary foci of cobalt epileptic rats. The mechanism of action was proposed to be inhibition of GABA-T activity in the brain; however, the protective effect of AOAA was more pronounced at the lower dose (5 mg/kg) while the enhancement of CNS GABA-T levels was more pronounced at higher doses, where the functional benefit of AOAA was less pronounced.	[413]
Memory consolidation in male Sprague-Dawley rats	25 mg/kg/day i.p. for 8 days	In the shuttlebox shock avoidance used, controls animals showed learning both within and across sessions, while AOAA-treated only showed learning within sessions but exhibited a lack of consolidation across sessions. Because GABA plays a role in memory consolidation, the mechanism was hypothesized to relate to the inhibitory effect of AOAA on GABA-T, but no pharmacological mechanism was investigated in the study.	[414]
Hyperbaric oxygen induced seizures in chicken	2.5 mg/kg s.q. single dose	AOAA decreased the onset and duration of the convulsions. The mechanism of action was proposed to be inhibition by AOAA of the GABA-T activity in the CNS and an elevation of central GABA levels, but no biochemical markers were measured.	[415]
Dichlorovinylcysteine induced nephrotoxicity model in male NMRI mice	40 mg/kg i.p. single dose	AOAA attenuated the generation of various lipid peroxidation markers. The mechanism of action was not directly explored but was presumed to be related to an antioxidant effect of AOAA.	[416]
Circulating glucose and insulin and glucagon levels in control and streptozotocin-diabetic female Wistar rats	30 mg/kg i.p. single dose	In control animals, AOAA significantly increased circulating insulin levels (but not glucose or glucagon levels). In the diabetic animals, AOAA protected against the development of streptozotocin-induced hyperglycemia. Streptozotocin caused a 50% drop in plasma insulin levels in the rats; this effect was largely absent in the AOAA-treated streptozotocin animals. The proposed mechanism relates to AOAA’s effect on some peripheral GABA-T system and subsequent increases in peripheral GABA levels, but no direct measurements were provided.	[417]
Male Wistar rats subjected to stroke (transient middle cerebral artery occlusion)	2.5, 5, 10 or 50 mg/kg i.p. single dose	AOAA at 10 and 50 mg/kg significantly reduced stroke volume and brain edema and improved neurological scores, without affecting post-ischemic cerebral blood flow, brain malondialdehyde content, SOD, or glutathione peroxidase activity. The mechanism of action was proposed to be inhibition of CBS activity by AOAA in the brain, but no biochemical markers were measured.	[418]
Hypoxia-induced central apneas in ventilated C57BL/6J mice	30 mg/kg i.p. single dose	AOAA reduced the percentage of animals expressing one or more apneas during reoxygenation. AOAA-treated mice also exhibited a smaller coefficient of variation for frequency during reoxygenation, suggesting improved respiratory stability. The mechanism of action was proposed to be inhibition of CBS activity in the CNS, but no biochemical markers were measured.	[419]
Cisplatin nephrotoxicity in male C57BL/6 mice or F344 rats	100 mg/kg p.o., single dose	AOAA protected against the biochemical (plasma BUN) and histological (renal tubular alterations) damage induced by cisplatin. The mechanism of action was proposed to be inhibition of cysteine S-conjugate b-lyase activity by AOAA (and/or an inhibitory effect of AOAA on some other PLP-dependent enzyme, most likely a transaminase). However, no experiments were conducted to delineate the molecular mechanism of AOAA’s action.	[420,421]
Tumor growth in female BALB/c nude mice bearing MDA-MB-231 human breast cancer subcutaneous xenografts	10 mg/kg/day i.p. for 14 days	AOAA significantly inhibited tumor growth. Based on complementary in vitro studies, the mechanism of AOAA’s action was proposed to relate to the suppression of tumor cell bioenergetics, in particular due to AOAA-mediated inhibition of tumor cell aspartate aminotransferase activity (an enzyme which functions in tandem with malate dehydrogenase to regulate mitochondrial electron transport).	[394]
Complete Freund adjuvant (CFA)-induced mechanical hyperalgesia model in adult Sprague-Dawley rats	5, 15 or 45 mg/kg/day i.p. single dose	AOAA dose-dependently attenuated mechanical hyperalgesia due to an inhibition of the hyperexcitability of dorsal root ganglion neurons. In these neurons, CFA up-regulated CBS mRNA transcription and subsequent translation of CBS protein. The mode of AOAA’s action was proposed to be related to inhibition of CBS activity, and the consequent prevention of the H_2_S-mediated opening of tetrodotoxin-resistant voltage-gated sodium channels.	[422]
Tumor growth in female athymic nude mice bearing subcutaneous xenografts of HCT116 colon cancer cells or human patient-derived colon cancer xenografts (PDTX). Liver metastasis model (nude mice, intracecal HCT116 implantation)	1, 3 or 9 mg/kg/day i.p. for 2 weeks	AOAA (at 9 mg/kg/day, but not at the lower doses) suppressed tumor growth. The underlying mechanisms was proposed to relate to the AOAA-induced inhibition of intratumor CBS, inhibition of intratumor H_2_S production, which, in turn, inhibits cellular bioenergetics and reduces tumor angiogenesis. The effect of AOAA was independent of the tumor’s K-ras status. The effects of AOAA were reproduced by the AOAA prodrug YD0171, which, however, was more potent (effective at 0.5 and 1 mg/kg/day). YD0171 (at 3 mg/kg/day for 3 weeks), caused the regression of established HCT116 subcutaneous xenografts. YD0171 also inhibited liver metastasis formation in an intracecal HCT116 implantation model.	[88,296,320]
Athymic Balb/c mice bearing SUM149, SUM159, or HCC1954 MDA-MB-231 xenografts; MMTV-rTtA-TetO-myc mouse mammary tumor model	5 mg/kg/day i.p. or 0.5 mg/kg/day i.p. in the TetO-myc model	AOAA suppressed the growth of the UM149, SUM159 xenografts, but did not affect the growth of HCC1954 xenografts. AOAA was also effective in the TetO-myc model. In the MDA-MB-231 xenografts, AOAA did not inhibit tumor growth alone, but potentiated the growth-suppressant effect of paclitaxel. The underlying mechanisms was proposed to relate to the inhibition of intratumor GOT activity, as it is associated with increased C-MYC expression in the tumors and the subsequent increased reliance of the tumor cells on glutaminolysis.	[369]
Male BALB/c mice subjected to burn injury	10 mg/kg/day i.p. for 6 days	AOAA attenuated the degree of burn-induced oxidative stress in various tissues. It also reduced plasma levels of various circulating mediators (IL-6, IL-10). It improved various plasma markers of multiorgan failure. The effects were attributed to AOAA’s effect as an inhibitor of CBS.	[175]
Female athymic nude mice bearing subcutaneous xenografts of various human colon cancer tumor lines	5 or 10 mg/kg/day i.p. for 2–4 weeks (depending on the growth of the particular cell line graft)	AOAA dose-dependently reduced tumor growth of the HCT116, DLD1, RKO, and HT29 xenografts, but did not affect the growth of SW40 or LoVo xenografts). The underlying mechanisms was proposed to relate to the inhibition by AOAA of glutamate pyruvate transaminase 2 (GPT2) in the tumor cells. This hypothesis was supported by the findings that the growth of PIK3CA mutant xenograft tumors (which express GPT2) were inhibited by AOAA, but GPT2 knockdown tumors were not. (It should be noted, however that the latter tumors showed a significantly slower baseline proliferation rate in the absence of AOAA).	[423]
Male Wistar rats subjected to experimental subarachnoid hemorrhage induced by double blood injection; effect of L-cysteine	5 mg/kg i.p. single dose	AOAA suppressed the neuroprotective effect of L-cysteine. Its mechanism of action was proposed to be inhibition of CBS-induced H_2_S production. The authors’ working hypothesis is that L-cysteine increases CBS-derived H_2_S production, and this produces neuroprotective effects. Unfortunately, the effect of AOAA (in the absence of L-cysteine) was not tested in the study.	[424]
Female athymic nude mice bearing subcutaneous xenografts of NCM356 colon epithelial cells overexpressing CBS	9 mg/kg/day i.p. for 2 weeks	AOAA significantly decreased the size of established tumors. The underlying mechanisms was proposed to relate to the inhibition of intratumor CBS activity by AOAA and the consequent inhibition of intratumor H_2_S production. Metabolomic and pharmacological studies also implicated a role for the pentose phosphate pathway in the CBS-mediated enhancement of tumor growth.	[303]
Experimental allergic encephalomyelitis model in C57BL/6 mice induced by a myelin oligodendrocyte glycoprotein peptide fragment	35 mg/kg/day i.p. for 7 days	Disease severity was suppressed by AOAA. The effect of AOAA was associated with significant changes in immune cell populations. The percentage of IL-17-producing T cells was reduced while the percentage of FOXP3+ T cells increased, while the percentage of IFNγ + cells was unaffected in the central nervous system. The ratio of FOXP3+ cells to IL-17+ cells increased by AOAA. AOAA markedly reduced the total number of mononuclear cells infiltrating into the central nervous system. Based on complementary in vitro and in vivo studies, the mechanism proposed to underlie AOAA’s action was proposed to relate to the suppression of immune cell bioenergetics, in particular due to AOAA-mediated inhibition of GOT1 activity, which produces an increase in 2-hydroxyglutarate levels in differentiating TH17 cells, which in turn results in the hypermethylation of the Foxp3 gene locus and inhibited Foxp3 transcription, which ultimately regulates the differentiation towards TH17.	[178]
Male Sprague-Dawley rats subjected to an experimental model of chronic alcoholism (chronic ethanol consumption)	5 mg/kg/day i.p. for 2 weeks	Alcoholism produced learning and memory deficits (assessed by the Morris water maze test). AOAA improved latency and swimming distance parameters and improved the animals’ performance in the spatial probe test. AOAA also prevented the down-regulation of myelin basic protein expression and protected against the deterioration of mitochondrial ultrastructure. The mechanism of action was proposed to be inhibition of CBS activity by AOAA in the brain; the AOAA-induced normalization of hippocampal H_2_S levels provided some experimental support for this theory. AOAA also induced complex changes in gene expression and antioxidant levels in the brain of the animals.	[425,426]
Male Swiss albino mice subjected to stroke (transient middle cerebral artery occlusion) in combination with remote ischemic preconditioning	50 mg/kg i.p. single dose	AOAA suppressed the neuroprotective effect of remote ischemic preconditioning. Its mechanism of action was proposed to be inhibition of CBS-induced H_2_S production. The authors’ working hypothesis is that stroke down-regulates CBS expression in the CNS, and this down-regulation is prevented by preconditioning. Unfortunately, the effect of AOAA on stroke (in the absence of preconditioning) was not tested in the study.	[427]
Male and female SOD1G93A mice, a model of familial ALS	8.75 mg/kg/day i.p. for 100 days	AOAA significantly improved motor performance (Rotarod test) in the female (but not male) animals and tended to extend survival. The underlying mechanisms was proposed to relate to an up-regulation of CBS in ALS, which, in turn, elevates H_2_S to cytotoxic concentrations. Thus, it was hypothesized that inhibition of CBS activity with AOAA reduces neuronal and glial H_2_S levels to physiological (cytoprotective) levels. The gender difference was proposed to relate to higher levels of CNS H_2_S levels in females with ALS than males with ALS.	[428]
Male athymic nude mice bearing subcutaneous human colon cancer cell line xenografts	9 mg/kg/day i.p. 5 days per week for 4 weeks	AOAA potentiated the inhibitory effect of oxaliplatin on tumor growth, but on its own, did not exert a significant inhibitory effect. The underlying mechanisms was proposed to relate to the AOAA-induced inhibition of intratumor CBS and the subsequent inhibition of intratumor H_2_S production, with a consequent suppression of cellular bioenergetics and of tumor angiogenesis. The potentiation of oxaliplatin’s antitumor effect was hypothesized to be related to an enhancement by AOAA of oxaliplatin-induced tumor cell apoptosis.	[318]

i.p. = intraperitoneal administration; i.v. = intravenous administration; p.o. = per os (oral administration); s.q. = subcutaneous administration.

**Table 4:** AOAA-inhibitable enzymes, listed in chronological order (publications showing that these enzymes are inhibited by AOAA). The references represent the initial discovery and the (often subsequent) determination of IC_50_ and/or Ki values. ^a^ The published data on aspartate aminotransferase inhibition parameters are controversial. Braunstein (1973) reported a low K_i_, which is consistent with the fact that AOAA is usually described as a potent aspartate aminotransferase inhibitor. However, Rofe (1978), reports an IC_50_ of 170 µM. The table presents the value published by Braunstein. ^b^ Aspartate aminotransferase and cysteine aminotransferase is the same protein catalyzing two different reactions (quite common for PLP-dependent enzymes). However, there are no published biochemical reports studying the kinetic of AOAA-mediated CAT inhibition. Therefore, the same value is presented for both. ^c^ In some cases, neither IC_50_ nor K_i_ values are available in the literature; in such instances, the AOAA concentration is presented at which full inhibition of the enzyme was reported.

Classification	Enzyme	IC_50_ (µM)	K_i_ (µM)	Reference
EC 2.6.1.19	4-aminobutyrate-2-oxoglutarate transaminase (GABA-T)	1.8		[353,371,430]
EC 2.6.1.2	alanine transaminase (ALT) (aka glutamate pyruvate transaminase, GPT) (aka alanine:oxalacetate transaminase)	0.5		[431,432]
EC 2.6.1.6	D-amino acid transaminase	0.1		[433]
EC 2.7.1.35	pyridoxal kinase	10		[434]
EC 4.1.1.22	histidine decarboxylase	5		[357,435]
EC 4.1.1.19	arginine decarboxylase	500		[435]
EC 4.2.1.22	cystathionine β-synthase (CBS)	3–8		[329,363,399,437]
EC 2.6.1.1	aspartate transaminase (AST) ^a^ (aka glutamic oxaloacetic transaminase, GOT)		<0.1	[405,438,439]
EC 2.6.1.3	cysteine transaminase (CAT) ^b^		<0.1	[405,438,439]
EC 4.1.2.5	threonine aldolase	1000–5000		[440]
EC 2.1.2.1	serine hydroxymethyltransferase	1000		[441,442]
EC 5.1.1.1	alanine racemase	>10		[358,443]
EC 4.4.1.9	beta-cyano-L-alanine synthase	10–100		[444]
EC 4.4.1.1	cystathionine γ-lyase (CSE)	1		[364,399]
EC 4.3.1.5	phenylalanine ammonia-lyase	0.45		[445]
EC 2.6.1.7	kynurenine-oxoglutarate transaminase	25		[445,446]
EC 4.1.1.20	diaminopimelate decarboxylase	2500 ^c^		[447]
EC 2.6.1.42	branched-chain amino acid transaminase		21	[383,448]
EC 2.6.1.5	tyrosine transaminase	∼20		[449]
EC 4.1.1.28	DOPA decarboxylase	50 ^c^		[362,450]
EC 2.6.1.44	alanine-glyoxylate transaminase	0.15		[451]
EC 4.4.1.14	1-aminocyclopropanecarboxylate synthase		0.8	[452]
EC 4.4.1.4	alliin lyase	250 ^c^		[453]
EC 4.4.1.15	D-cysteine desulfhydrase		3.3	[400,454]
EC 4.1.1.11	cysteine sulfinic acid decarboxylase	30		[455,456,457]
EC 2.6.1.45	serine-glyoxylate aminotransferase	0.01		[442,458]
EC 2.6.1.37	(2-aminoethyl)phosphonate-pyruvate transaminase	1000 ^c^		[459]
EC 2.6.1.43	aminolevulinate transaminase	0.03		[460]
EC 4.1.1.12	L-aspartate 4-carboxy-lyase		1.6	[461]
EC 4.4.1.13	cysteine-S-conjugate beta-lyase (mitochondrial)		8	[462]
EC 4.4.1.13	cysteine-S-conjugate beta-lyase (cytosolic)		0.8	[462]
EC 4.1.1.15	glutamate decarboxylase	1		[463,464]
EC 5.1.1.18	serine racemase	∼100		[465]

**Table 5:** Pharmacological inhibitors of CBS. Compounds set in bold letters are discussed, in detail, in Section 5. Please note that different experimental conditions yield different relative IC_50_ values for CBS inhibition. For instance, CBS activity and the inhibitory potency of various compounds are influenced by the source and structure of the recombinant CBS used (i.e., species, full length vs. truncated etc.), as well as by the assay conditions (e.g., pH, buffer, time of pre-incubation of test compounds with the enzyme, read-out of the assay, plate format, etc.). As an example, a slight modification in the composition of the assay buffer produced a shift in the IC_50_ of hydroxylamine from 20 µM to 400 µM [328]. There is also the possibility that the inhibitory effects reported are, at least in part, related to reactions with H_2_S (in the assays where H_2_S production was used to assess CBS activity) and/or interferences with the fluorescent probe used (e.g., quenching). Such effects have been documented, for instance, for copper (which directly reacts with and decomposes H_2_S) and for several polyphenols and natural compounds that have emerged from the CBS screening campaigns [327,329,571]. The selectivity of the inhibitors for CBS vs. CSE is discussed as well (whenever data are available). Since the structure and the catalytic mechanism of the third H_2_S-producing enzyme, 3-MST is different, it is generally not expected that the compounds shown herein would act as significant inhibitors of 3-MST. However, the potential effect of these compounds on 3-MST has not been tested. Conversely, inhibitors of 3-MST identified from screening campaigns would not be expected to inhibit CBS or CSE. Nevertheless, from a set of 3-MST inhibitors identified by Hanaoka and colleagues [572], some of them actually exhibit some CBS-inhibitory effect as well, while others are apparent CBS and CSE activators.

Inhibitor Structure	Name	IC_50_	Selectivity	Reference
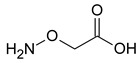	Aminooxyacetic acid	1–8.5 μM	The compound is a potent CBS inhibitor which works by reacting with its PLP prosthetic group. Although it is commonly referred to as a “CBS inhibitor”, it is an even more potent inhibitor of CSE (IC_50_: 1 µM)	[296,302,329]
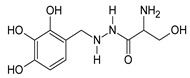	Benserazide	30 μΜ	Relatively potent CBS inhibitor that reacts with its PLP prosthetic group. It has some selectivity for CBS (CSE is inhibited 16% at 100 µM benserazide and 3-MST is inhibited 50% at 300 µM benserazide)	[327,329]
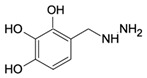	2,3,4-Trihydroxy-benzylhydrazine	30 μΜ	It inhibits CBS by reacting with its PLP prosthetic group. It may be responsible for some of the CBS-inhibitory effect of benserazide in vivo. Its effect on CSE has not been tested	[329]
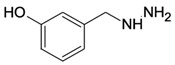	3-Hydroxy-benzylhydrazine	60 µM	It inhibits CBS by reacting with its PLP prosthetic group. Its effect on CSE has not been tested. It is known to inhibit GABA-T and other PLP-dependent enzymes	[329]
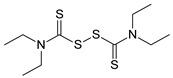	Disulfiram	Not a direct inhibitor	In yeast assays and in Down syndrome mice, it has biological effects consistent with cell-based CBS inhibition	[259]
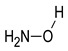	Hydroxylamine	20–400 μM	The compound inhibits CBS, but it inhibits CSE more potently (IC_50_: 5 µM)	[328]
Cu^2+^	Copper	0.2–10 µM	The assessment of true CBS-inhibitory potency is made difficult by the fact that it also reacts with H_2_S, the product of the CBS reaction measured in the assay	[329,542,543]
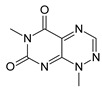	NSC 67078 1,6-dimethyl-pyrimido[5,4-e]-1,2,4-triazine-5,7(1*H*,6*H*)-dione	12–30 µM	It preferentially inhibits CBS; it also inhibits CSE, but with lower potency (IC_50_: 30 µM)	[328,329]
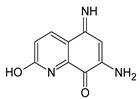	NSC11041	4 µM	Approximately equipotent on inhibitor of CBS and CSE (IC_50_ ~3–4 µM)	[328]
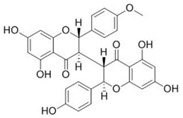	Sikokianin C	3.1 µM	Potent CBS inhibitor; its potency on CSE is weaker (IC_50_: 40 µM)	[302,562]
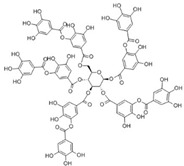	Tannic acid	40 µM	CBS inhibitor; its effect on other H_2_S producing enzymes has not been tested	[329]
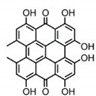	Hypericin	3.1 µM	Potent CBS inhibitor; its potency on CSE is weaker (IC_50_: 40 µM)	[562]
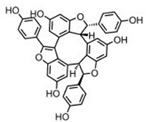	Caraphenol A	5.9 µM	Fairly potent CBS inhibitor; its potency on CSE is almost comparable (IC_50_: 12 µM)	[562]
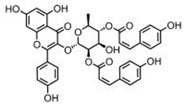	2′′,4′′-Di-*O*-(*Z*-p-coumaroyl) afzelin	6.2 µM	Potent CBS inhibitor; its potency on CSE is very weaker (IC_50_ > 400 µM)	[562]
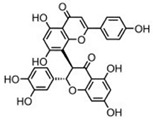	3′-Hydroxy-volkensiflavon	7.8 µM	Potent CBS inhibitor; its potency on CSE is very weaker (IC_50_ > 400 µM)	[562]
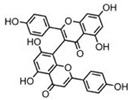	Cupressuflavone	11.5 µM	Potent CBS inhibitor; its potency on CSE is very weaker (IC_50_ > 400 µM)	[562]
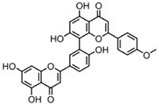	Podocarpusflavone A	8.9 µM	Potent CBS inhibitor; its potency on CSE is very weaker (IC_50_ > 400 µM)	[562]
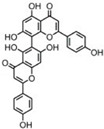	Agathisflavone	17.1 µM	Potent CBS inhibitor; its potency on CSE is very weaker (IC_50_ > 400 µM)	[562]
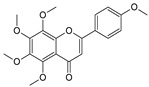	Tangeritin	IC_25_: 46 µM	CBS inhibitor; its effect on other H_2_S producing enzymes has not been tested	[327]
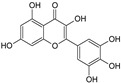	Myricetin	18.8 µM	Fairly potent CBS inhibitor; its potency on CSE is similar (IC_50_: 14.4 µM)	[562]
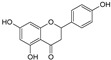	Apigenin	83 µM	CBS inhibitor; its effect on other H_2_S producing enzymes has not been tested	[327]
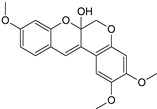	12α-hydroxy-5-deoxydehydro-munduserone	56 µM	CBS inhibitor; its effect on other H_2_S producing enzymes has not been tested	[327]
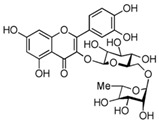	Rutin	116 µM	CBS inhibitor; its effect on other H_2_S producing enzymes has not been tested	[327]
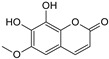	Fraxetin	134 µM	CBS inhibitor; its effect on other H_2_S producing enzymes has not been tested	[327]
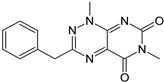	CH004	0.6–1.7 µM	A highly potent CBS inhibitor, with some selectivity towards CBS over CSE (IC_50_ ~ 30 µM)	[256]
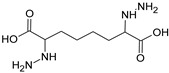	6S	Ki = 48 µM	It inhibits CBS inhibitor via interacting with its PLP group. Its effect on other H_2_S-producing enzymes or other PLP-dependent enzymes has not been characterized	[566]
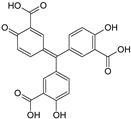	Aurintricarboxylic acid	3–80 µM	CBS inhibitor with considerable potency; it is even more potent as a CSE inhibitor (IC_50_ 0.6–3 µM)	[329,570]
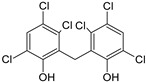	Hexachlorophene	60 µM	CBS inhibitor with average potency	[329]
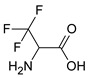	Trifluoroalanine	66 μΜ	It does not have a high potency as a CBS inhibitor, but it exhibits some selectivity for CBS over CSE (IC_50_ ~ 300 µM)	[399]
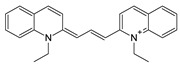	JHU-8555	8–12 µM	Approximately equipotent on inhibitor of CBS and CSE, with some preference for CBS (IC_50_ ~ 10–25 µM)	[328]
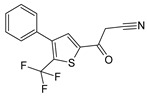	MBSEW03275	15 µM	It does not have a high inhibitory potency as a CBS inhibitor, but it does have some selectivity for CBS over CSE (IC_50_ ~ 200 µM)	[328]
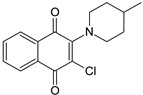	SP14311008	20 µM	Approximately equipotent on inhibitor of CBS and CSE, with some preference for CBS (IC_50_ ~ 40 µM for CSE)	[328]
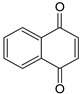	1,4-Naphtoquinone	35 µM	CBS inhibitor; its effect on other H_2_S producing enzymes has not been tested	[327]
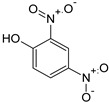	2,4-Dinitrophenol	56 µM	CBS inhibitor; its effect on other H_2_S producing enzymes has not been tested	[327]
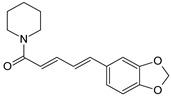	Piperine	61 µM	CBS inhibitor; its effect on other H_2_S producing enzymes has not been tested	[327]
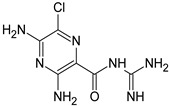	Amiloride	89 µM	CBS inhibitor; its effect on other H_2_S producing enzymes has not been tested	[327]
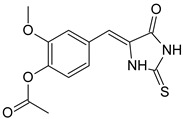	MNP2-A6	83 µM	CBS inhibitor; its effect on other H_2_S producing enzymes has not been tested	[571]
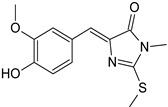	MNP2-B7	87 µM	CBS inhibitor; its effect on other H_2_S producing enzymes has not been tested	[571]
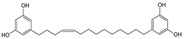	NP-014428	7.4 µM	Fairly potent CBS inhibitor; its potency on CSE is weaker (IC_50_: 62 µM)	[562]
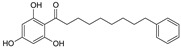	NP-003872	8.1 µM	Fairly potent CBS inhibitor; its potency on CSE is weaker (IC_50_: 122 µM)	[562]
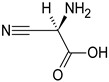	β-cyano-alanine	40% inhibition at 10 mM	The compound is a weak CBS inhibitor, but it is a potent inhibitor of CSE (IC_50_: 14 µM)	[399]
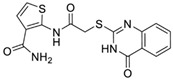	“Compound #1” 3-MST inhibitor	25% inhibition at 100 µM	The compound was identified as a potent 3-MST inhibitor (IC_50_: 1.7 µM), but it also exerts a weak inhibitory effect on CBS and CSE	[572]
